# The binding of Class II sRNA MgrR to two different sites on matchmaker protein Hfq enables efficient competition for Hfq and annealing to regulated mRNAs

**DOI:** 10.1261/rna.067777.118

**Published:** 2018-12

**Authors:** Joanna Kwiatkowska, Zuzanna Wroblewska, Kenneth A. Johnson, Mikolaj Olejniczak

**Affiliations:** 1 Institute of Molecular Biology and Biotechnology, Faculty of Biology, Adam Mickiewicz University, 61-614 Poznań, Poland; 2 Institute of Cellular and Molecular Biology, University of Texas, Austin, Texas 78712, USA

**Keywords:** Hfq, MgrR, *eptB*, *ygdQ*, Class II sRNA

## Abstract

MgrR is an Hfq-dependent sRNA, whose transcription is controlled by the level of Mg^2+^ ions in *Escherichia coli*. MgrR belongs to Class II sRNAs because its stability in the cell is affected by mutations in Hfq differently than canonical, Class I sRNAs. Here, we examined the effect of mutations in RNA binding sites of Hfq on the kinetics of the annealing of MgrR to two different target mRNAs, *eptB* and *ygdQ*, by global data fitting of the reaction kinetics monitored by gel electrophoresis of intermediates and products. The data showed that the mutation on the rim of the Hfq ring trapped MgrR on Hfq preventing the annealing of MgrR to either mRNA. The mutation in the distal face slowed the ternary complex formation and affected the release of MgrR-mRNA complexes from Hfq, while the mutation in the proximal face weakened the MgrR binding to Hfq and in this way affected the annealing. Moreover, competition assays established that MgrR bound to both faces of Hfq and competed against other sRNAs. Further studies showed that uridine-rich sequences located in less structurally stable regions served as Hfq binding sites in each mRNA. Overall, the data show that the binding of MgrR sRNA to both faces of the Hfq ring enables it to efficiently anneal to target mRNAs. It also confers on MgrR a competitive advantage over other sRNAs, which could contribute to efficient cellular response to changes in magnesium homeostasis.

## INTRODUCTION

Small regulatory RNAs contribute to bacterial response to environmental stress, maintenance of cell homeostasis (Updegrove et al. 2015; Wagner and Romby 2015), and the modulation of bacterial virulence (Ellis et al. 2017; Heidrich et al. 2017). Small RNAs (sRNAs) act by pairing with partly or fully complementary sequences in regulated mRNAs to affect their transcription, translation, or stability (Bobrovskyy and Vanderpool 2016; Hao et al. 2016; Sedlyarova et al. 2016; Chao et al. 2017; Gorski et al. 2017). The regulation by sRNAs is often dependent on accessory proteins (Holmqvist and Vogel 2018), such as Hfq (Updegrove et al. 2016; Kavita et al. 2018), ProQ (Olejniczak and Storz 2017) or Crc (Hernández-Arranz et al. 2016). The matchmaker protein Hfq, which is homologous to eukaryotic Sm proteins, is involved in the regulation by numerous *trans*-encoded sRNAs in *Escherichia coli*, *Salmonella enterica*, and other enterobacteria (Holmqvist et al. 2016; Melamed et al. 2016).

Magnesium ion homeostasis in bacterial cells is regulated by the PhoP/PhoQ two-component system (Groisman et al. 2013). This system controls numerous genes involved in Mg^2+^ uptake (Wang et al. 2017) and cell surface modification (Moon et al. 2013). MgrR is a PhoP/PhoQ-dependent sRNA, and its transcription is induced in response to moderately low Mg^2+^ and Ca^2+^ levels (Moon and Gottesman 2009). MgrR was proposed to regulate numerous mRNA targets, but it had the largest effects on the levels of *eptB* mRNA, which encodes a lipopolysaccharide modification enzyme, and *ygdQ* mRNA, which encodes an inner membrane protein of unknown function (Moon and Gottesman 2009; Moon et al. 2013; Lee and Gottesman 2016; Melamed et al. 2016).

The homohexameric, ring-shaped Hfq serves as a platform for simultaneous binding of sRNAs and regulated mRNAs to facilitate their annealing (Updegrove et al. 2016). Additionally, Hfq rearranges the structures of interacting RNAs to enable the pairing of complementary sequences (Soper and Woodson 2008; Soper et al. 2011; Wroblewska and Olejniczak 2016). To simultaneously engage different RNAs, Hfq uses three RNA binding sites on the proximal and distal faces of its ring, and on the rim (Mikulecky et al. 2004; Link et al. 2009; Sauer et al. 2012). Despite limiting Hfq concentrations in the cell and tight RNA binding, sRNAs are rapidly exchanged on Hfq, which enables Hfq to efficiently participate in numerous interactions (Fender et al. 2010; Hussein and Lim 2011; Moon and Gottesman 2011; Olejniczak 2011). The mechanism of sRNA exchange is based on competitor-induced dissociation and involves acidic C-terminal domains of Hfq (Wagner 2013; Santiago-Frangos et al. 2016).

The majority of sRNAs, called canonical or Class I sRNAs, recognize the proximal and rim surfaces of Hfq, while their target mRNAs bind to the distal face (Mikulecky et al. 2004; Sauer et al. 2012; Schu et al. 2015). The proximal face of Hfq binds the 3′-terminal oligouridine tails of these sRNAs (Otaka et al. 2011; Sauer and Weichenrieder 2011; Ishikawa et al. 2012; Morita et al. 2017), while the positively charged patches on the rim of Hfq contact the adenosine and uridine-rich sequences present in the central or 5′-terminal regions of sRNA molecules (Sauer et al. 2012; Dimastrogiovanni et al. 2014). The distal face of Hfq recognizes adenosine-rich motifs, including repeated ARN sequences, which are present in mRNAs regulated by these sRNAs (de Haseth and Uhlenbeck 1980; Mikulecky et al. 2004; Soper and Woodson 2008; Link et al. 2009; Soper et al. 2011; Peng et al. 2014a,b; Wroblewska and Olejniczak 2016; Chen and Gottesman 2017; Andrade et al. 2018). Hence, the canonical sRNAs and their mRNA targets bind to the opposite faces of Hfq, which enables Hfq to promote their annealing.

Beyond the canonical sRNAs a distinct, smaller group of sRNAs, called Class II, was recently described based on the different dependence of their stability in the cells on mutations in Hfq (Zhang et al. 2013; Schu et al. 2015). MgrR belongs to this group together with ChiX, CyaR, and McaS sRNAs. The lifetimes of sRNAs from this group were increased when Hfq had a mutation in the rim surface, which suggested that the rim was involved in binding to regulated mRNAs. It was opposite to what was observed for canonical sRNAs, which were stabilized by the distal face mutation, consistent with the binding of their mRNA targets to the distal face (Schu et al. 2015).

To better understand how Hfq contributes to MgrR sRNA pairing with regulated mRNAs, here we analyzed the kinetics of MgrR annealing to *eptB* and *ygdQ* mRNAs in the presence of Hfq mutants in vitro. Additionally, we used structure probing to analyze the context of Hfq binding sites in MgrR and in *eptB* and *ygdQ* mRNAs. Finally, the effects of mutations in uridine-rich sequences of these two mRNAs were studied to better understand their recognition by Hfq. The results further elucidated the interactions of MgrR sRNA with Hfq and provided support for the hypothesis that there is a range of alternative sRNA and mRNA recognition modes by the matchmaker protein Hfq, which enables it to efficiently promote the pairing of different RNAs.

## RESULTS

### The repeated ARN sequence motif present in MgrR is located in a single-stranded region

**FIGURE 1.**
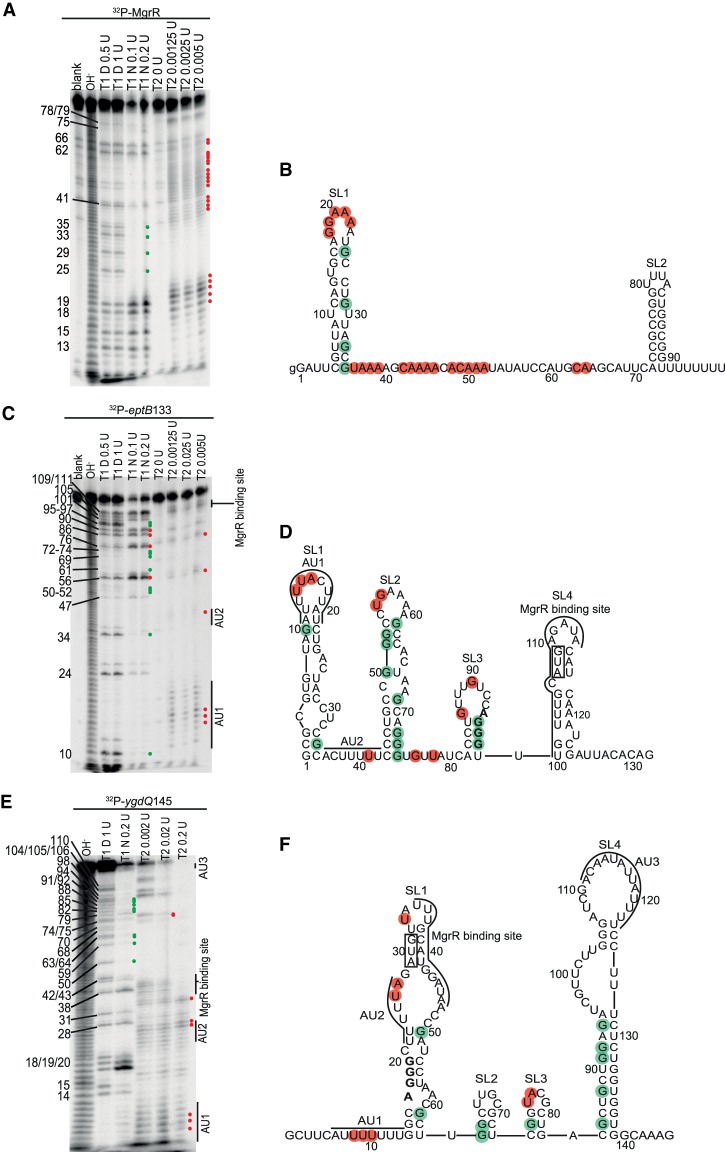
Secondary structures of *E. coli* MgrR sRNA and 5′-terminal fragments of mRNAs *eptB* and *ygdQ*. (*A*) The probing of ^32^P-MgrR structure with RNases indicated *above* the lanes. (*B*) The secondary structure of MgrR predicted by *RNAstructure* software based on data from probing experiments in *A* and in Supplemental Figure S1A. (*C*) The probing of ^32^P-*eptB*133 mRNA structure with RNases indicated *above* the lanes. (*D*) The secondary structure of *eptB*133 mRNA predicted by *RNAstructure* software based on data from probing experiments in *C* and in Supplemental Figure S1B. (*E*) The probing of ^32^P-*ygdQ*145 mRNA structure with RNases indicated *above* the lanes. (*F*) The secondary structure of *ygdQ*145 mRNA predicted by *RNAstructure* software based on data from probing experiments in *E* and in Supplemental Figure S1C. Symbols T1 D and T1 N denote probing with RNase T1 in denaturing or native conditions, respectively, while T2 denotes probing with RNase T2. The numbers to the *left* indicate positions of guanosine residues. Blank denotes untreated control and OH^−^ denotes formamide ladder. Positions of the residues susceptible to degradation by RNase T2, Nuclease S1 (Supplemental Fig. S1A), or RNase T1 in native conditions were constrained as single-stranded (red circles) in secondary structure prediction using *RNAstructure* software (Reuter and Mathews 2010), while positions protected from RNase T1 cleavage in native conditions as compared to the denaturing conditions were constrained as double-stranded (green circles). MgrR binding sites and the locations of AU-rich sequence motifs are marked with lines in *D* and *F*. The AUG start codon is boxed and SD sequences are marked in bold font.

To understand the impact of the RNA structure on the Hfq contribution to MgrR annealing to its targets, MgrR sRNA was probed with structure-specific RNases (Fig. 1A,B; Supplemental Fig. S1A). The sites of degradation induced by RNase T2 and Nuclease S1 were used to determine single-stranded regions, while the comparison of RNase T1 cleavages in native conditions versus denaturing conditions defined the double-stranded regions. The nucleotide positions determined as single-stranded or double-stranded by probing with RNases were used as constraints in structure prediction using *RNAstructure* software (Reuter and Mathews 2010). The probing data showed that two stable stem–loop motifs were formed in the 5′- and 3′-terminal regions of MgrR, while the central part of the molecule was likely poorly structured. The protection of guanosine 41 from cleavage by RNase T2 and Nuclease S1 could suggest its involvement in some transient secondary structure interactions or alternatively, it could reflect the sequence specificity of cleavage by these RNases. As it has been previously observed (Schu et al. 2015), the 98-nt-long MgrR sRNA contains in its central part a long adenosine-rich sequence, which could also be considered as an (ARN)_5_ motif with two mismatches (Fig. 1A,B). Most of this sequence is susceptible to cleavage by both RNase T2 and Nuclease S1, which suggests it could be accessible for binding by Hfq. The region from U55 to A68, which is involved in MgrR pairing to several regulated mRNAs, including *eptB* and *ygdQ* mRNAs (Moon and Gottesman 2009), is also located in this central, single-stranded region. The cleavage pattern of the 3′-terminal U_8_ sequence was not well resolved, but this region was also predicted to be single-stranded. The fact that adenosine-rich and uridine-rich sequences of MgrR are not involved in stable secondary structure is consistent with their proposed roles in binding of MgrR to the Hfq protein (Zhang et al. 2013; Schu et al. 2015).

### The binding of MgrR involves interactions with the opposite surfaces of Hfq

To better understand how MgrR is recognized by Hfq, we used a gelshift assay to measure the binding of the sRNA to wild-type (wt) Hfq and various mutants: K56A mutation in the proximal face, Y25D mutation in the distal face, or R16A mutation in the rim (Supplemental Fig. S2). The data showed that MgrR bound wt Hfq with an equilibrium dissociation constant (*K*_d_) value of 0.5 nM (Supplemental Fig. S2). Similar *K*_d_ values were previously determined for other *E. coli* sRNAs in equilibrium experiments using filter retention (Olejniczak 2011; Małecka et al. 2015). Subnanomolar *K*_d_ values were also determined for binding of MicA sRNA and *ompA* mRNA to Hfq using surface plasmon resonance (Fender et al. 2010), and the *K*_d_ values in the low nanomolar range were obtained for RNA-OUT sRNA (Ross et al. 2013), and DsrA, RyhB, RprA, and ChiX sRNAs in equilibrium experiments using gelshift assays (Santiago-Frangos et al. 2016). The affinity of MgrR to the distal face (Y25D) mutant and to the rim (R16A) mutant of Hfq was also in the subnanomolar range, while the affinity of MgrR to the proximal face (K56A) mutant was weaker than to the wt Hfq (Supplemental Fig. S2). This effect is different than observed previously for ChiX sRNA, where the binding was not affected by the K56A mutation, which could be explained by tighter binding of ChiX than MgrR to the opposite, distal face of Hfq (Małecka et al. 2015). The detrimental effect of the proximal face mutation is explained by the fact that the 3′-terminal oligouridine tails of sRNA molecules interact specifically with the binding pockets on the proximal face of Hfq (Sauer and Weichenrieder 2011). This observation is also consistent with the in vivo data, which showed that the Hfq proximal face mutations resulted in lower accumulation and consistently higher turnover of MgrR, as well as other sRNAs, suggesting weakened binding to Hfq (Schu et al. 2015). Hence, these data suggest that the binding of the 3′-terminal U_8_ tail of MgrR to the proximal face provides the biggest contribution to the stability of the binary complex of MgrR and Hfq.

**FIGURE 2.**
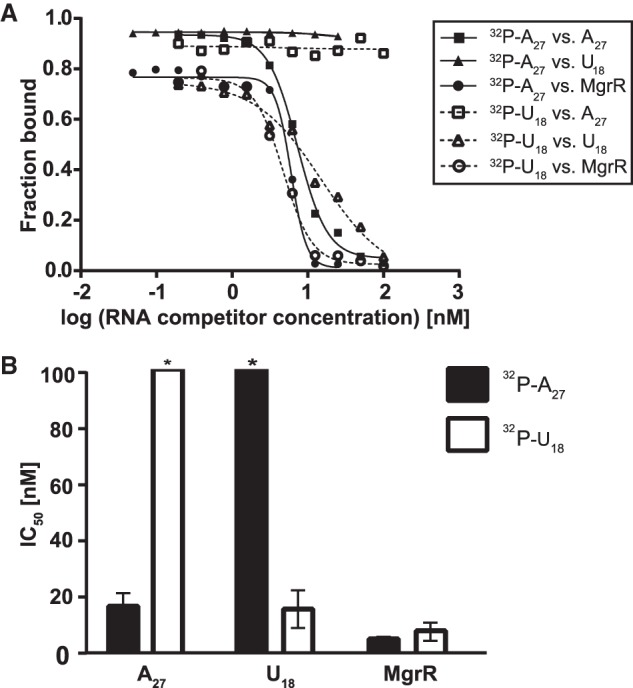
MgrR efficiently displaces both A_27_ and U_18_ from the complex with Hfq. (*A*) The dissociation of ^32^P-A_27_ or ^32^P-U_18_ from Hfq, which was initiated by unlabeled MgrR sRNA, A_27_, or U_18_, was monitored by a double-filter retention assay. The data were fit to the three-parameter equation for unlabeled A_27_ and U_18_ and and to the four-parameter equation for unlabeled MgrR sRNA, as described in Materials and Methods. (*B*) The average IC_50_ values for the competition against ^32^P-A_27_ or ^32^P-U_18_. The average values of Hill coefficients in the fitting were −3.2 ± 1.2 for MgrR competition against ^32^P-A_27_, and −3.3 ± 1.8 for MgrR competition against ^32^P-U_18_. Bars marked with (*) correspond to IC_50_ values, which were estimated as higher than 100 nM.

To obtain a more detailed picture of the interactions between MgrR and Hfq, the competition of MgrR against ^32^P-labeled oligourydylate U_18_ and oligoadenylate A_27_ was compared (Fig. 2). Oligourydylate is expected to preferably bind the proximal face (Olejniczak 2011; Sauer and Weichenrieder 2011), while oligoadenylate the distal face of Hfq (Mikulecky et al. 2004; Link et al. 2009). The data showed that MgrR efficiently displaced both A_27_ and U_18_ from the complex with Hfq (Fig. 2). This effect is the same as previously observed for the competition of another Class II sRNA ChiX against the same oligonucleotides, while it is in contrast to the behavior of the Class I sRNA RybB, which efficiently displaced U_18_, but was inefficient in competition against A_27_ (Małecka et al. 2015). These data suggest that even though the binding of MgrR to Hfq is more strongly dependent on the contacts with the proximal face of Hfq (Supplemental Fig. S2), MgrR interacts with both the proximal and the distal face of Hfq (Fig. 2).

**FIGURE 3.**
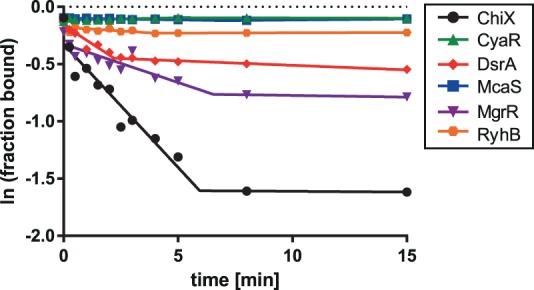
Comparison of sRNAs in competition for binding to Hfq. The dissociation of ^32^P-MgrR sRNA from Hfq was initiated by adding unlabeled sRNA competitors at 5 nM concentration. The fitting of the data using segmental linear regression provided $k_{\mathrm{off}}^{1}$ and $k_{\mathrm{off}}^{2}$ values of, respectively, 250 × 10^−4^ sec^−1^ and 4 × 10^−4^ sec^−1^ for ChiX, 7.2 × 10^−4^ sec^−1^ and 0.64 × 10^−4^ sec^−1^ for CyaR, 170 × 10^−4^ sec^−1^ and 14 × 10^−4^ sec^−1^ for DsrA, 5 × 10^−4^ sec^−1^ and 2.9 × 10^−4^ sec^−1^ for McaS, 110 × 10^−4^ sec^−1^ and 4.7 × 10^−4^ sec^−1^ for MgrR, 37 × 10^−4^ sec^−1^ and 0.29 × 10^−4^ sec^−1^ for RyhB. Average *k*_off_ values are presented in Table 2.

To analyze the competition of MgrR against other sRNAs the rates of displacement of ^32^P-MgrR from Hfq, induced by six different sRNAs, were compared (Table 1; Fig. 3). For this purpose, a previously described competition assay based on filter retention was used (Małecka et al. 2015). The data showed that only ChiX sRNA displaced MgrR from Hfq with a rate twofold faster than that induced by unlabeled MgrR itself, while DsrA was similarly efficient as MgrR (Table 1; Fig. 3). Three other sRNAs, which were CyaR, McaS, and RyhB, were less efficient in competition. Similar efficiencies of MgrR and ChiX were also observed in previous in vivo studies of competition against the regulation of *rpoS* by DsrA, RprA, and ArcZ sRNAs (Moon and Gottesman 2011). The better competition efficiency of MgrR than RyhB was also shown in these in vivo studies. On the other hand, these studies showed similar competition efficiency of MgrR and CyaR (Moon and Gottesman 2011), which suggests a more complex behavior of some sRNAs in the competition in vivo.

**TABLE 1.**
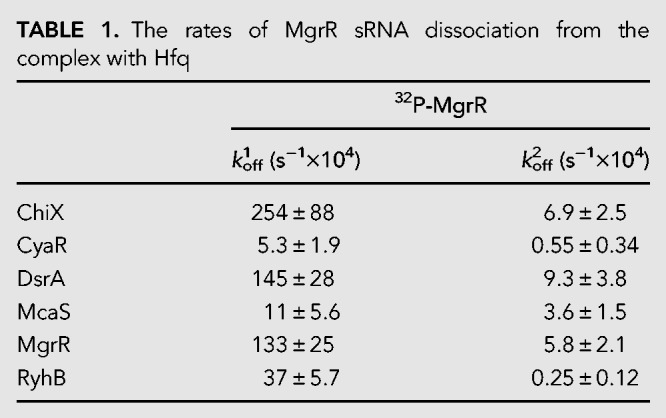
The rates of MgrR sRNA dissociation from the complex with Hfq

### The 5′-terminal regions of *eptB* and *ygdQ* mRNAs contain unstructured adenosine- and uridine-rich sequences

To investigate how Hfq recognizes mRNAs regulated by MgrR the structures of 5′-terminal fragments of *eptB* and *ygdQ* mRNAs were probed with RNases T1 and T2 (Fig. 1C–F; Supplemental Fig. S1B,C). A 133-nt-long 5′-terminal fragment of *eptB* mRNA, named *eptB*133, was selected for this study (Fig. 1D), because previous data showed that it was sufficient for translation regulation by MgrR when fused to β-galactosidase reporter gene in vivo (Moon et al. 2013). This region contains two uridine-rich sequences, the simultaneous mutation of which was detrimental for repression of *eptB* mRNA translation by MgrR (Schu et al. 2015). The 13-nt-long 5′-AU_4_ACU_2_AUCU-3′ sequence, named AU1, is located at nucleotide positions 11–23 of 5′-UTR, while the 7-nt-long 5′-ACU_5_-3′ sequence, named AU2, is located at positions 36–42. The patterns of degradation suggested that *eptB*133 consists of four stem–loop motifs (SL1–SL4) (Fig. 1C,D; Supplemental Fig. S1B). Both uridine-rich sequences were located in single-stranded regions. The AU1 sequence was located in the loop of SL1, and the AU2 sequence in the linker between SL1 and SL2. The SL2 structure was resistant to cleavage, except for the apical loop region, which was consistent with its stabilization by several G-C pairs. The short SL3 motif was stabilized by a four-base-pair stem including a Shine–Dalgarno sequence. Finally, the MgrR pairing site, which overlaps the AUG start codon, is located in the adjacent stem–loop SL4. As it mostly contains A-U pairs it is likely to be structurally dynamic, which would facilitate its unfolding for pairing with MgrR.

For the probing of the structure of the *ygdQ* mRNA, we used a 5′-terminal 145-nt-long fragment, named *ygdQ*145 (Fig. 1E,F; Supplemental Fig. S1C). Although the MgrR pairing site is located in the 5′-terminal part of this fragment, control experiments showed that MgrR did not anneal efficiently to a shorter 83-nt-long 5′-terminal fragment of *ygdQ* mRNA (data not shown), presumably because of mRNA misfolding, and hence, the longer 145-nt sequence was used. The MgrR pairing site is located at the beginning of the *ygdQ* coding sequence. The 5′-terminal region of *ygdQ* contains two uridine-rich regions upstream of the MgrR binding site: an AU_7_ sequence (named AU1, nucleotide positions 6–13), and an U_5_A sequence (named AU2, positions 22–27). Additionally, a 13-nt-long adenosine- and uridine-rich sequence (named AU3, positions 111–123) is located downstream from the MgrR binding site. The data showed that an ∼40-nt-long region, including AU1 and AU2 sequences, on the 5′-terminus of *ygdQ* mRNA is susceptible to cleavage by RNase T2. This suggests that the predicted SL1, which includes this region, is structurally dynamic (Fig. 1E,F). The central part of *ygdQ*145 is predicted to fold into two short stem–loops, and it is adjacent to an extended SL4, which includes the AU3 sequence in its apical loop (Fig. 1E,F). Hence, similarly to *eptB*, the 5′-terminal fragment of *ygdQ* mRNA contains several AU-rich sequences located in structurally dynamic regions, which could serve as Hfq binding sites.

### Hfq stimulates the annealing of MgrR to *eptB* and *ygdQ* mRNAs

**FIGURE 4.**
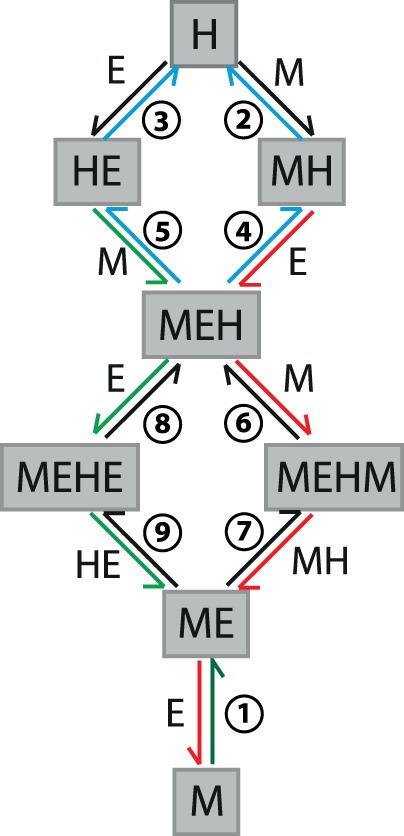
The scheme of the reactions used to model the progress of MgrR annealing to *eptB*133 and *ygdQ*145. The numbers correspond to the following reactions: (1) M + E = ME; (2) M + H = MH; (3) H + E = HE; (4) MH + E = MEH; (5) HE + M = MEH; (6) MEH + M = MEHM; (7) ME + MH = MEHM; (8) MEH + E = MEHE; (9) ME + HE = MEHE, where M is MgrR, E is mRNA (either *eptB*133 or *ygdQ*145 in respective reactions), ME is binary complex of MgrR with respective mRNA, MH is binary complex of MgrR with Hfq, HE is binary complex of respective mRNA with Hfq, MEH is a ternary complex of Hfq with MgrR and respective mRNA, MEHE is a complex of Hfq with MgrR and two molecules of respective mRNA, MEHM is a complex of Hfq with respective mRNA and two molecules of MgrR. Colored arrows (blue, green, red) mark reactions included in three closed thermodynamic loops that were constrained during the data fitting to the model.

**FIGURE 5.**
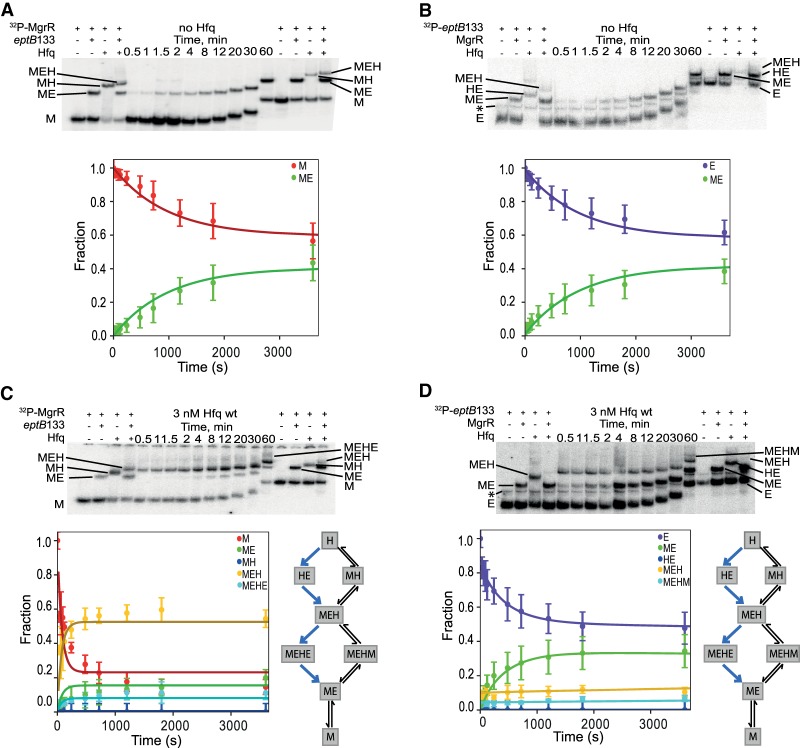
Global modeling of MgrR annealing to *eptB*133 in the presence of wt Hfq. The native gel analysis of the kinetics of (i) ^32^P-labeled MgrR annealing to *eptB*133 in the absence of Hfq (*A*) or the presence of Hfq (*C*), and (ii) ^32^P-labeled *eptB*133 annealing to MgrR in the absence of Hfq (*B*) or the presence of wt Hfq (*D*). The data from *A*, *B*, *C*, and *D* were analyzed globally, and the lines in the plots are the results of global fitting. The schemes on the *right* of the plots in *C* and *D* describe kinetically preferred competing pathways (blue arrows) based on the data from Table 4. Unbound MgrR is marked as M, unbound *eptB*133 as E, MgrR-*eptB*133 complex as ME, MgrR-Hfq complex as MH, *eptB*133-Hfq complex as HE, and MgrR-*eptB*133-Hfq ternary complex as MEH. MEHE and MEHM denote quaternary complexes formed in excess of unlabeled *eptB*133 or MgrR, respectively. The asterisk indicates a slower migrating band, which is interpreted as the dimeric form of ^32^P-*eptB*133.

**FIGURE 6.**
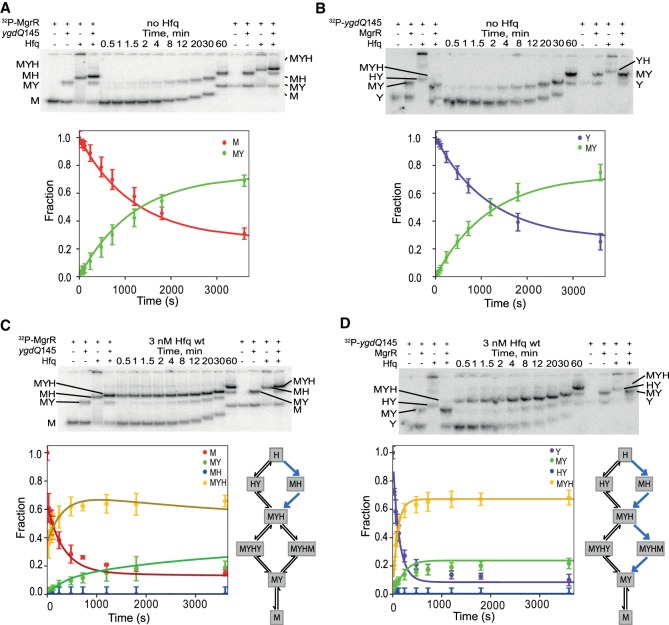
Global modeling of MgrR annealing to *ygdQ*145 in the presence of wt Hfq. The native gel analysis of the kinetics of (i) ^32^P-labeled MgrR annealing to *ygdQ*145 in the absence of Hfq (*A*) or the presence of wt Hfq (*C*), and (ii) ^32^P-labeled *ygdQ*145 annealing to MgrR in the absence of Hfq (*B*) or the presence of wt Hfq (*D*). The data from *A*, *B*, *C*, and *D* were analyzed globally, and the lines in the plots are the results of global fitting. The schemes on the right of the plots in *C* and *D* describe kinetically preferred competing pathways (blue arrows) based on the data from Table 4. Unbound MgrR is marked as M, unbound *ygdQ*145 as Y, MgrR-*ygdQ*145 complex as MY, MgrR-Hfq complex as MH, *ygdQ*145-Hfq complex as HY, and MgrR-*ygdQ*145-Hfq ternary complex as MYH.

**FIGURE 7.**
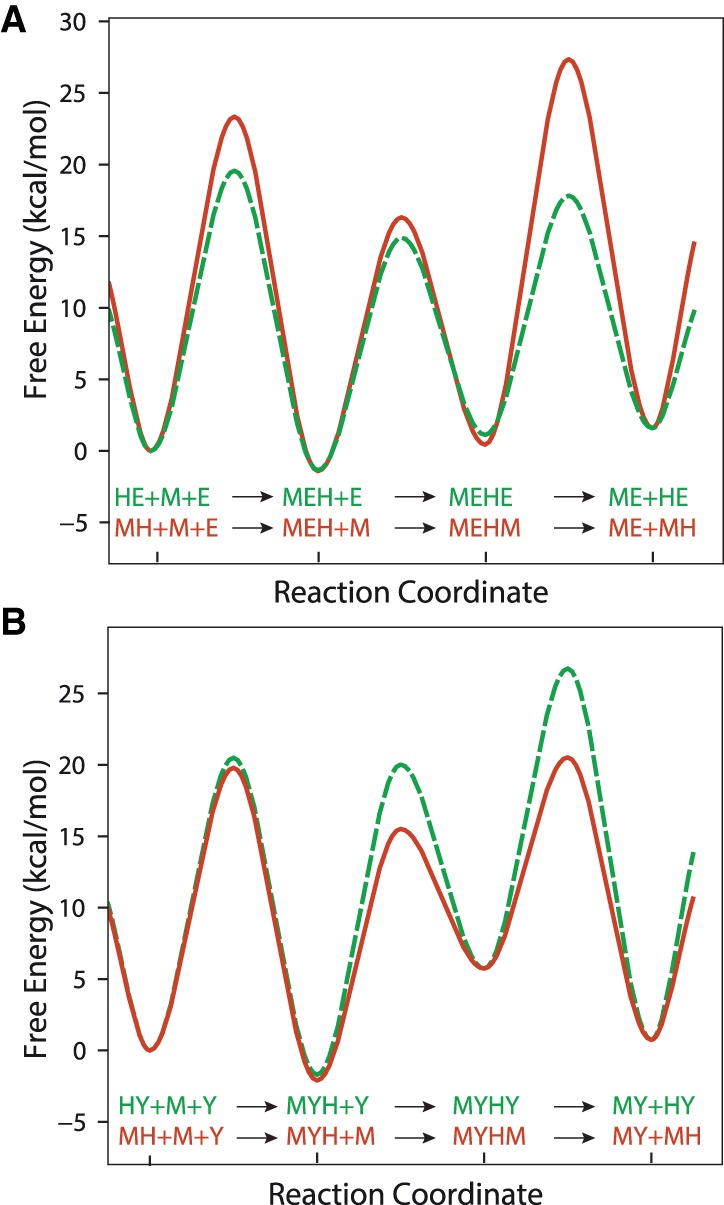
Free energy profiles for the annealing of MgrR to *eptB*133 or *ygdQ*145 mRNA fragments. The free energy profiles for the competing pathways in the annealing of MgrR to *eptB*133 mRNA (*A*) or *ygdQ*145 mRNA (*B*) were computed with *KinTek Explorer* using simple transition state theory (see Materials and Methods). The symbols describing unbound molecules and their complexes are the same as in the legends to Figures 5 and 6 for reactions with *eptB*133 and *ygdQ*145, respectively.

**FIGURE 8.**
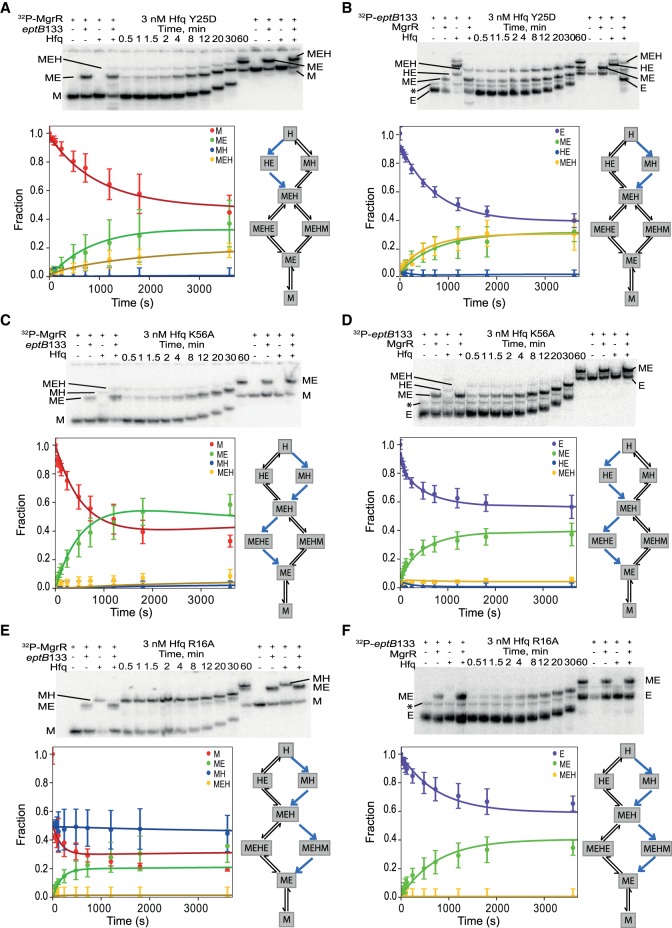
Global modeling of MgrR annealing to *eptB*133 in the presence of Hfq variants with mutations in the distal, proximal, and rim RNA binding sites. The native gel analysis of the kinetics of (i) ^32^P-labeled MgrR annealing to *eptB*133 in the presence of Hfq Y25D (*A*), Hfq K56A (*C*), or Hfq R16A (*E*), and (ii) ^32^P-labeled *eptB*133 annealing to MgrR in the presence of Hfq Y25D (*B*), Hfq K56A (*D*), or Hfq R16A (*F*). The data for each of the Hfq mutants were analyzed globally with the data for the annealing in the absence of Hfq (Fig. 5A,B) (see Materials and Methods), and the lines in the plots are the results of global fitting. The schemes on the *right* of each plot describe kinetically preferred competing pathways (blue arrows) based on the data from Table 4. The RNAs and their complexes are marked in the same way as in Figure 5.

**FIGURE 9.**
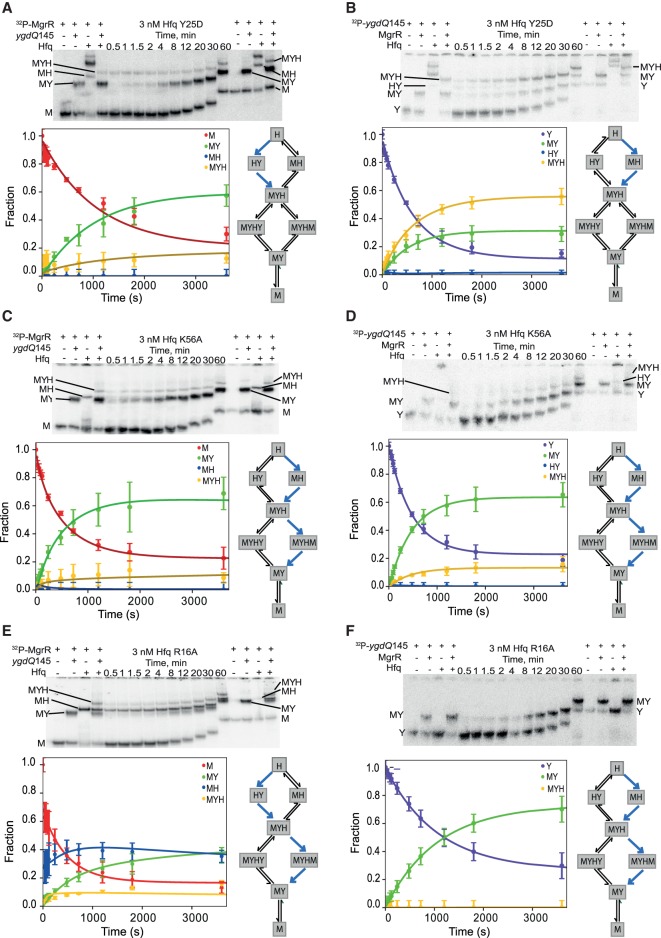
Global modeling of MgrR annealing to *ygdQ*145 in the presence of Hfq variants with mutations in the distal, proximal, and rim RNA binding sites. The native gel analysis of the kinetics of (i) ^32^P-labeled MgrR annealing to *ygdQ*145 in the presence of Hfq Y25D (*A*), Hfq K56A (*C*), or Hfq R16A (*E*) and (ii) ^32^P-labeled *ygdQ*145 annealing to MgrR in the presence of Hfq Y25D (*B*), Hfq K56A (*D*), or Hfq R16A (*F*). The data for each of the Hfq mutants were analyzed globally with the data for the annealing in the absence of Hfq (Fig. 6A,B) (see Materials and Methods), and the lines in the plots are the results of global fitting. The schemes on the *right* of each plot describe kinetically preferred competing pathways (blue arrows) based on the data from Table 4. The RNAs and their complexes are marked in the same way as in Figure 6.

To explain how Hfq affects the pairing of MgrR to *eptB* and *ygdQ* mRNAs their annealing was monitored using a gelshift assay followed by the global analysis of reaction kinetics using *KinTek Explorer* software (Johnson et al. 2009b; Tables 2–4; Figs. 4–9). The annealing of MgrR to each mRNA was studied when either MgrR or mRNA was labeled and the other RNA was in 100-fold excess, in the absence or presence of wt Hfq (Figs. 5, 6). Subsequently, the four data sets were analyzed together by fitting data globally to a model that consisted of nine reactions describing the direct annealing of MgrR to respective mRNA (reaction 1 in Fig. 4), the formation of the ternary complex of MgrR with mRNA and Hfq (reactions 2–5 in Fig. 4), and the release of the binary complex of MgrR with mRNA from the ternary complex with Hfq induced by the binding of another RNA (reactions 6–9 in Fig. 4). The data sets could not be well fit globally without invoking the binding of a third RNA molecule to the ternary complex (reactions 6 and 8 in Fig. 4) to stimulate the displacement of the annealed RNA from Hfq (reactions 7 and 9 in Fig. 4). When additionally the direct release of the duplex RNA from the ternary complex with Hfq was included in the model it did not affect the data fitting (data not shown). This reaction was neither necessary nor sufficient to account for the observed kinetics. Therefore, in refined data fitting, this step was deleted in order to simplify the model. Accordingly, our analysis suggested that the competing RNA is required for the displacement of the binary sRNA-mRNA complex from Hfq, and that it provides a driving force for stimulating the release of the binary complex thermodynamically as well as kinetically. This conclusion is consistent with the results of previous studies, which showed that *sodB* mRNA induced the dissociation of the binary RyhB-*sodB* complex from Hfq (Afonyushkin et al. 2005), and that several sRNAs and mRNAs could induce the dissociation of the binary MicA-*ompA* complex from Hfq (Fender et al. 2010). It is also consistent with data which showed that the rates of dissociation of sRNA molecules from Hfq were dependent on the concentrations of the competing RNAs (Fender et al. 2010; Olejniczak 2011; Wagner 2013).

**TABLE 2.**
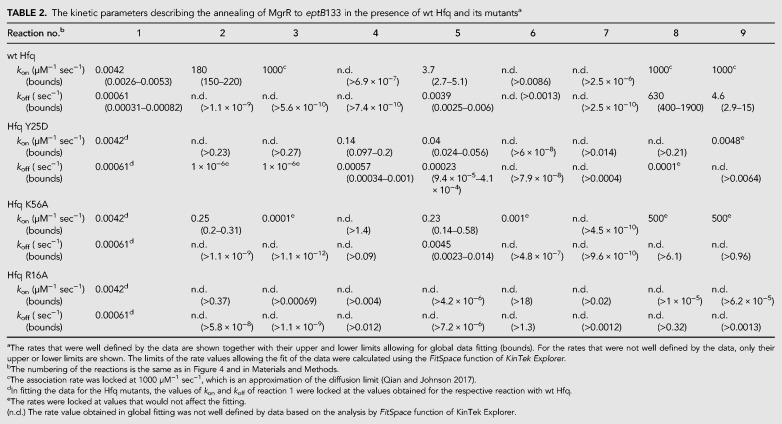
The kinetic parameters describing the annealing of MgrR to *eptB*133 in the presence of wt Hfq and its mutants^a^

**TABLE 3.**
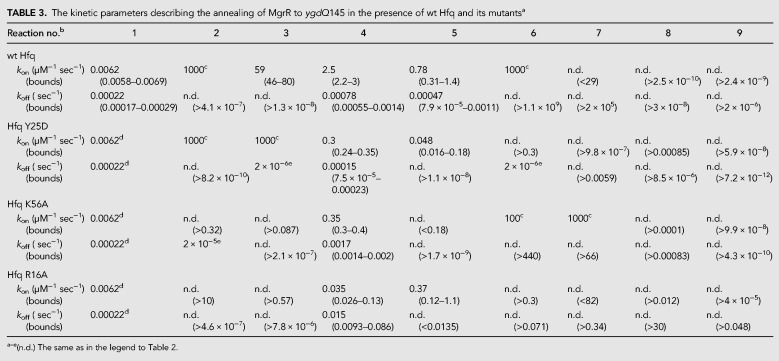
The kinetic parameters describing the annealing of MgrR to *ygdQ*145 in the presence of wt Hfq and its mutants^a^

**TABLE 4.**
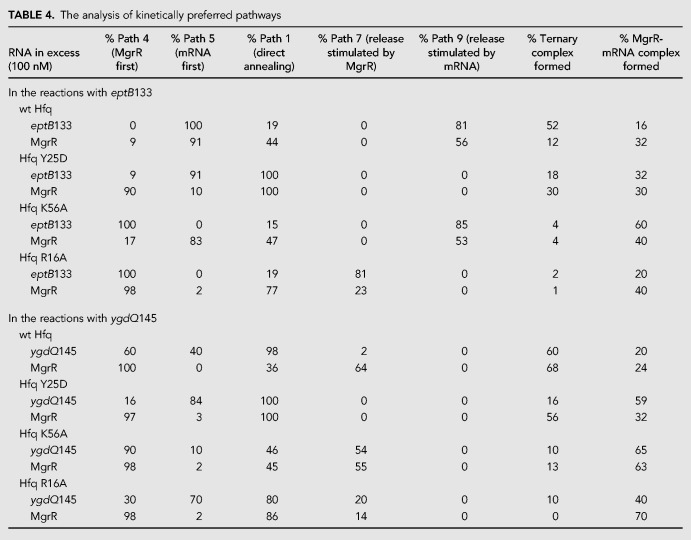
The analysis of kinetically preferred pathways

Although the model can account for the observed kinetics, there is insufficient information to define all of the rate constants. Rather, by modeling, we capture the essential features of the underlying pathway as represented by the model and a given set of rate constants. During global fitting, some rate constants continued toward extremely high or low values. However, in each case we could find a lower or upper limit, based on the confidence contour analysis with the *FitSpace* function of *KinTek Explorer*, beyond which the value of the rate constant did not affect the fitting (Johnson et al. 2009a). In these instances, we locked the rate constant at a value representing the threshold defining the upper or lower limit at which the rate constant did not affect the fitting (Tables 2, 3). The rate and equilibrium constants governing the direct annealing of MgrR to *eptB*133 or *ygdQ*145 were well defined by reactions performed in the absence of Hfq (Tables 2, 3; Figs. 5, 6). When the reactions in the presence and absence of Hfq were fit globally, the rate and equilibrium constants for direct annealing were used to enforce thermodynamic constraints so that the net free energy in forming the annealed RNA duplex in solution was the same in the presence or absence of Hfq. Also note that there are three closed thermodynamic loops in the model for the reactions in the presence of Hfq (Fig. 4), and each of these was constrained so that there was no net free energy change for going around the loop and returning to the starting state. Additionally, the data allowed us to calculate the kinetically preferred pathways characterizing the flow of reactants through competing pathways in the model (Table 4; Fig. 4), and to compute free energy profiles, in which an apparent free energy of activation was calculated from the estimated rate constants for two competing pathways: one starting and ending with the MgrR-Hfq complex and the other starting and ending with Hfq-mRNA complex (Fig. 7).

The annealing of MgrR to *eptB*133 was stimulated by Hfq (Tables 2, 4; Fig. 5). In the absence of Hfq, the direct binding of MgrR to *eptB*133 occurred with the rates of association (*k*_on_) and dissociation (*k*_off_) that corresponded to a *K*_d_ value of 150 nM (Table 2; Fig. 5A,B). In the presence of wt Hfq, MgrR mainly formed a binary complex with *eptB*133 and a ternary complex with *eptB*133 and the Hfq protein (Fig. 5C,D). Additionally, higher-order complexes formed during the reactions (Fig. 5C,D), which were interpreted as quaternary complexes; in the reaction with excess *eptB*133, a molecule of *eptB*133 was bound or, in the reaction with excess MgrR, the molecule of MgrR was bound to the ternary MgrR-*eptB*133-Hfq complex. We propose that these complexes form transiently to stimulate the release of the binary MgrR-*eptB*133 complexes from Hfq, and that they correspond to the quaternary complexes described as MEHE or MEHM, respectively, in the model used to globally fit the data (Fig. 4). The analysis of kinetically preferred pathways suggested that the ternary MgrR-*eptB*133-Hfq complex formed preferentially via the path that starts from the binding of *eptB*133 mRNA to Hfq followed by the binding of MgrR (reactions 3 and 5) (Table 4; Figs. 4, 5). The release of MgrR-*eptB*133 complex from the ternary complex with Hfq was also initiated by the binding of *eptB*133 (reactions 8 and 9). This is also illustrated by the free energy profile, which showed that the pathway in which *eptB*133 stimulated the release of MgrR-*eptB*133 from Hfq was preferred over the pathway dependent on MgrR (Fig. 7A). Importantly, the analysis showed that more than 40% of the binary MgrR-*eptB*133 complex in reaction with excess MgrR, and more than 80% in the reaction with excess *eptB*133, were formed by the release from the ternary complex with Hfq, as opposed to direct annealing (Table 4). Hence, these data provided direct, quantitative support for the role of Hfq in stimulating the annealing of MgrR to *eptB* mRNA.

The annealing of MgrR to *ygdQ* was also stimulated by Hfq (Table 3; Fig. 6). The rates of association and dissociation of MgrR and *ygdQ*145 mRNA in the absence of Hfq corresponded to a *K*_d_ value of 35 nM, which was fourfold tighter than for the annealing of MgrR to *eptB*133 mRNA. In the presence of wt Hfq, the ternary complex of MgrR with *ygdQ*145 and Hfq formed more efficiently than the binary MgrR-*ygdQ*145 complex (Table 4; Fig. 6). The higher-order complexes proposed to contain an additional molecule of MgrR or mRNA were barely detectable and could not be quantified (Fig. 6). The analysis of kinetically preferred pathways showed that the ternary complex was preferentially formed via the path initiated by the binding of MgrR to Hfq followed by binding of *ygdQ*145 (reactions 2 and 4) (Table 4; Figs. 4, 6). The release of the binary MgrR-*ygdQ*145 complex from Hfq was also initiated by the binding of MgrR (reactions 6 and 7). This is further illustrated by the free energy profile (Fig. 7B). Overall, these data indicated that even though Hfq efficiently stimulated the annealing of MgrR to both *eptB*133 and *ygdQ*145 the details of their interactions with Hfq could be different, which was suggested by the differences in the pathways of the formation of ternary complexes and of the release of MgrR-mRNA pairs from Hfq.

### The mutation in the distal face of Hfq affects the ternary complex formation and the release of MgrR-mRNA complexes from Hfq

To better understand how Hfq contributes to MgrR annealing to *eptB* and *ygdQ* mRNAs, the effects of mutations in the RNA binding sites of Hfq on the annealing of MgrR to each mRNA were studied. The Y25D mutation greatly weakens the RNA binding to the distal face of Hfq (Mikulecky et al. 2004). The ternary complex of MgrR with *eptB*133 and the Y25D Hfq mutant was formed less efficiently in the reaction with excess *eptB*133 and more efficiently in the reaction with excess MgrR, as compared to respective reactions in the presence of wt Hfq (Tables 2, 4; Figs. 5, 8A,B). The analysis of the kinetically preferred pathways showed that in the reaction with excess *eptB*133, the ternary complex formed mostly via Hfq binding first to *eptB*133, while in the reactions with excess MgrR via binding first to MgrR (Table 4); therefore, there was no preference for the pathway of ternary complex formation with the Hfq Y25D mutant. Finally, the analysis of the data suggested that the ternary complex with the Y25D Hfq mutant did not dissociate to release the MgrR-*eptB*133 complex (Table 4). Hence, the binary MgrR-*eptB*133 complex observed in both reactions with Hfq Y25D was formed only by direct annealing.

The ternary complex of MgrR with *ygdQ*145 and the Y25D Hfq mutant formed less efficiently both in the reaction with excess *ygdQ*145, and with excess MgrR, as compared to the corresponding reactions with wt Hfq (Tables 3, 4; Figs. 6, 9A,B). Consistently, the rates of ternary complex formation (reactions 4 and 5), obtained by global data fitting, were slower than for the wt Hfq (Table 3). Moreover, in the reactions with excess *ygdQ*145, the ternary complex formed via the path in which Hfq bound first to *ygdQ*145, while in the reaction with excess MgrR, the reaction proceeded via the path in which Hfq bound first to MgrR (Table 4). This was identical to the observations of corresponding reactions of MgrR annealing to *eptB*133. Finally, the analysis of the preferred kinetic pathways showed that the binary MgrR-*ygdQ*145 complex was not released from the ternary complex, which was also the same as observed for the corresponding reactions with *eptB*133 (Table 4).

In summary, these data suggest that the distal face of Hfq contributes to the correct orientation of interacting RNAs on Hfq, which explains lower efficiency and different preferred kinetic pathways of ternary complex formation in comparison to reactions with wt Hfq. Additionally, the fact that the Y25D mutation prevented the efficient release of the ternary complex from Hfq in the reactions with both *eptB*133 and *ygdQ*145 suggested that the distal face is the site of binding of competitor RNAs initiating the release of the binary MgrR-mRNA complexes from Hfq. The overall less efficient formation of the ternary complexes is consistent with shorter lifetime of MgrR in cells with the Y25D mutant, as this would lead to weaker protection of MgrR by Hfq from degradation by cellular RNases (Schu et al. 2015).

### The mutation in the proximal face of Hfq leads to inefficient formation of the ternary complex with MgrR and mRNA

In the presence of the K56A Hfq mutant, which prevents RNA binding to the proximal face (Supplemental Fig. S2; Mikulecky et al. 2004; Sauer and Weichenrieder 2011), the ternary complexes with MgrR and either *eptB*133 or *ygdQ*145 accumulated only in small amounts (Table 4; Figs. 8C,D, 9C,D). At the same time, the binary complexes of MgrR with each mRNA formed efficiently. Because of the low fractions of ternary complexes, the analysis of kinetics for the formation and dissociation of the ternary complex in each reaction is expected to be less accurate (Table 4). Nonetheless, the low amplitude for formation of the ternary complex is accounted for in the global data fitting (we fit both rates and amplitudes of the reactions) and provides information to help define the kinetically preferred pathway. The data show consistently that for reactions with both *eptB*133 and *ygdQ*145, the binary complexes of MgrR with each mRNA formed both by direct pairing and by the release from the ternary complex (Table 4). This suggests that the K56A Hfq mutant retains the ability to promote the MgrR annealing to each mRNA despite the weak binding of MgrR to Hfq K56A (Supplemental Fig. S2) and the low stability of the ternary complexes. The inefficient accumulation of the ternary complex in the presence of the proximal face K56A mutant (Tables 2–4; Figs. 8C,D, 9C,D) is consistent with the previous observation that another proximal face mutant (Hfq F42A) significantly decreased the lifetime of MgrR in the bacterial cells (Schu et al. 2015), which could be explained by the weaker protection of MgrR by Hfq.

### The rim mutation traps MgrR on Hfq and prevents its annealing to *eptB* and *ygdQ* mRNAs

The effect of the mutation in the rim of Hfq was very different from the effects induced by the other two Hfq mutants. The R16A mutation resulted in the accumulation of a high fraction of the binary complexes of MgrR with Hfq, while the ternary complexes including *eptB*133 were not detected and those including *ygdQ*145 accumulated to only about 10% (Figs. 8E,F, 9E,F). The binary complex of MgrR with the Hfq R16A mutant accumulated to about 50% in the reaction with ^32^P-MgrR and excess *eptB*133 (Fig. 8E), and to about 40% in the reaction with ^32^P-MgrR and excess *ygdQ*145 (Fig. 9E). In contrast, the binary complexes of each mRNA with this Hfq mutant were not detected in the reactions where either *eptB*133 or *ygdQ*145 were ^32^P-labeled (Figs. 8F, 9F). This shows that the ternary complex formation is prevented by the interference of the R16A mutation with mRNA binding to Hfq. Due to the low level of ternary complexes formed, the analysis of preferred pathways is less reliable; however, the data for the reactions with both mRNAs suggest that most of the binary MgrR-mRNA complexes were formed by direct annealing and that the release from the ternary complex was induced solely by binding of MgrR (Table 4). Hence, all these data support the role of the rim of Hfq as the binding site of *eptB* and *ygdQ* mRNAs. This was previously hypothesized as a possible explanation based on the analysis of Hfq and mRNA mutants in bacterial cells (Schu et al. 2015).

Overall, the data for reactions with both mRNAs suggest that the binding of MgrR to the R16A Hfq mutant not only did not facilitate, but actually prevented the annealing of MgrR to two different mRNA targets, by trapping them on the dysfunctional Hfq mutant. This effect is consistent with the increased MgrR stability in cells containing R16A Hfq mutant, which would result from the decreased MgrR annealing to *eptB* or *ygdQ* mRNA causing longer protection by Hfq, and preventing the decay of MgrR together with the repressed mRNA (Schu et al. 2015).

### Hfq recognizes uridine-rich sequences in *eptB* and *ygdQ* mRNAs

**FIGURE 10.**
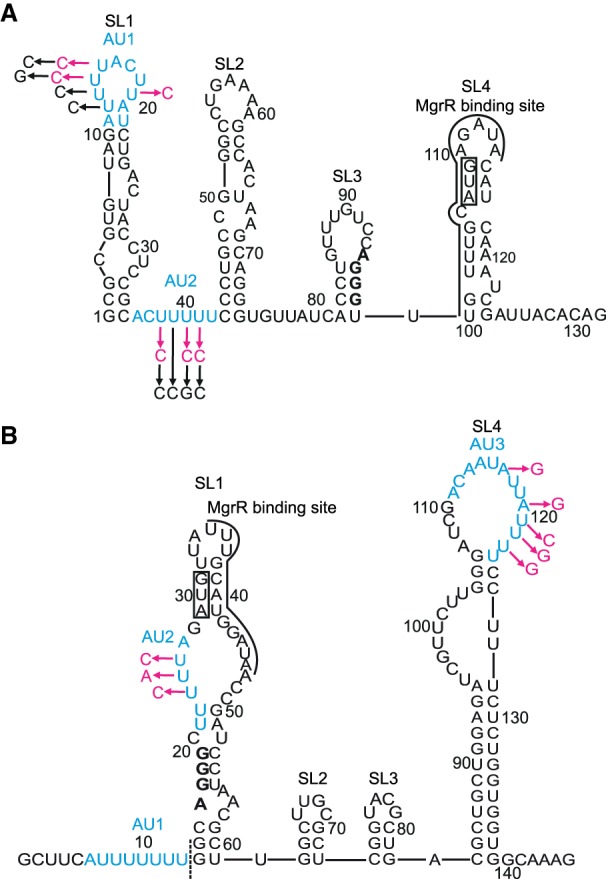
The mutations introduced into *eptB*133 and *ygdQ*145 mRNA fragments. Nucleotide substitutions (indicated with arrows) were introduced into the AU-rich sequences (blue letters) of (*A*) *eptB*133 and (*B*) *ygdQ*145 mRNAs. The mutations introduced into *eptB*133_AU1_v1 and *eptB*133_AU2_v1 mutants are marked with black font, and substitutions in other mutants of *eptB*133 and *ygdQ*145 are marked with pink font. The single-stranded region deleted on the 5′ end of *ygdQ*145_AU1 is marked by a dashed line. The AUG start codons are boxed and SD sequences are marked in bold font.

**FIGURE 11.**
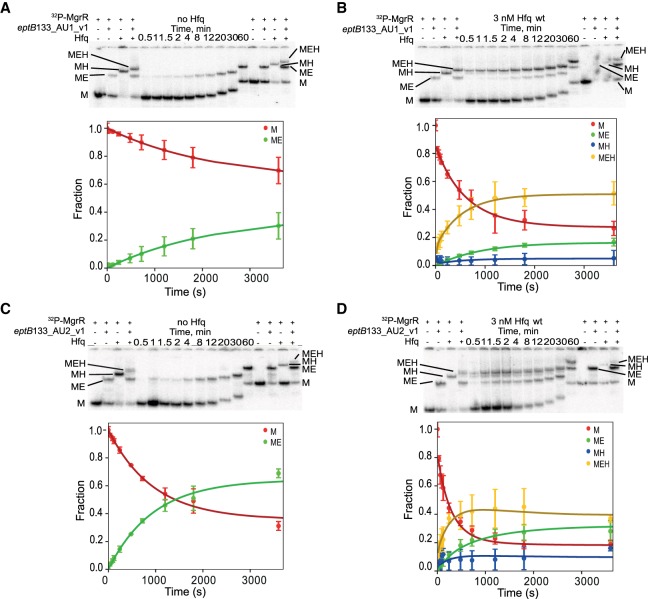
Global modeling of MgrR annealing to *eptB*133 with mutations in AU1 and AU2 sequence motifs (mutants *eptB*133_AU1_v1 and *eptB*133_AU2_v1). The native gel analysis of the kinetics of ^32^P-labeled MgrR annealing to (i) *eptB*133_AU1_v1 in the absence of Hfq (*A*) or the presence of Hfq (*B*) and (ii) *eptB*133_AU2_v1 in the absence of Hfq (*C*) or the presence of Hfq (*D*). The data for the annealing of MgrR to each of the mRNA mutants in the absence and presence of wt Hfq were analyzed globally, and the lines in the plots are the results of global fitting. The RNAs and their complexes are marked in the same way as in Figure 5.

**FIGURE 12.**
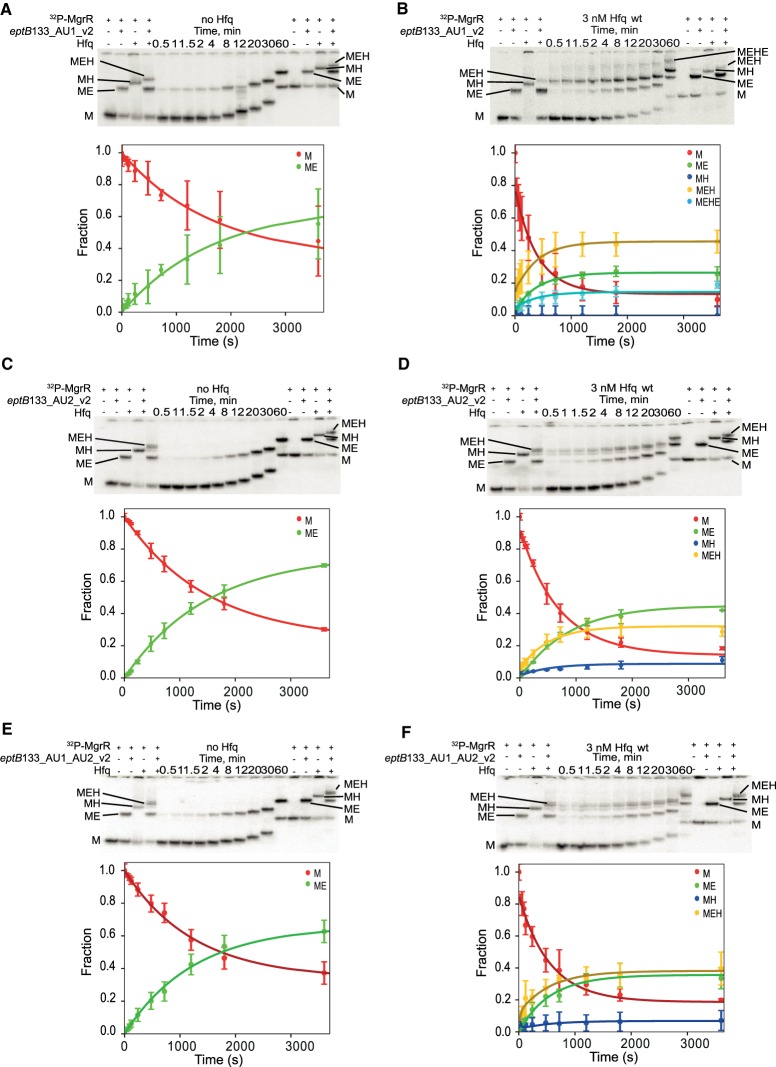
Global modeling of MgrR annealing to *eptB*133 with mutations in AU1 and AU2 sequence motifs (mutants *eptB*133_AU1_v2, *eptB*133_AU2_v2, and *eptB*133_AU1_AU2). The native gel analysis of the kinetics of ^32^P-labeled MgrR annealing to (i) *eptB*133_AU1_v2 in the absence of Hfq (*A*) or the presence of Hfq (*B*), (ii) *eptB*133_AU2_v2 in the absence of Hfq (*C*) or the presence of Hfq (*D*), and (iii) *eptB*133_AU1_AU2 in the absence of Hfq (*E*) or the presence of Hfq (*F*). The data for the annealing of MgrR to each of the mRNA mutants in the absence and the presence of wt Hfq were analyzed globally, and the lines in the plots are the results of global fitting. The RNAs and their complexes are marked in the same way as in Figure 5.

**FIGURE 13.**
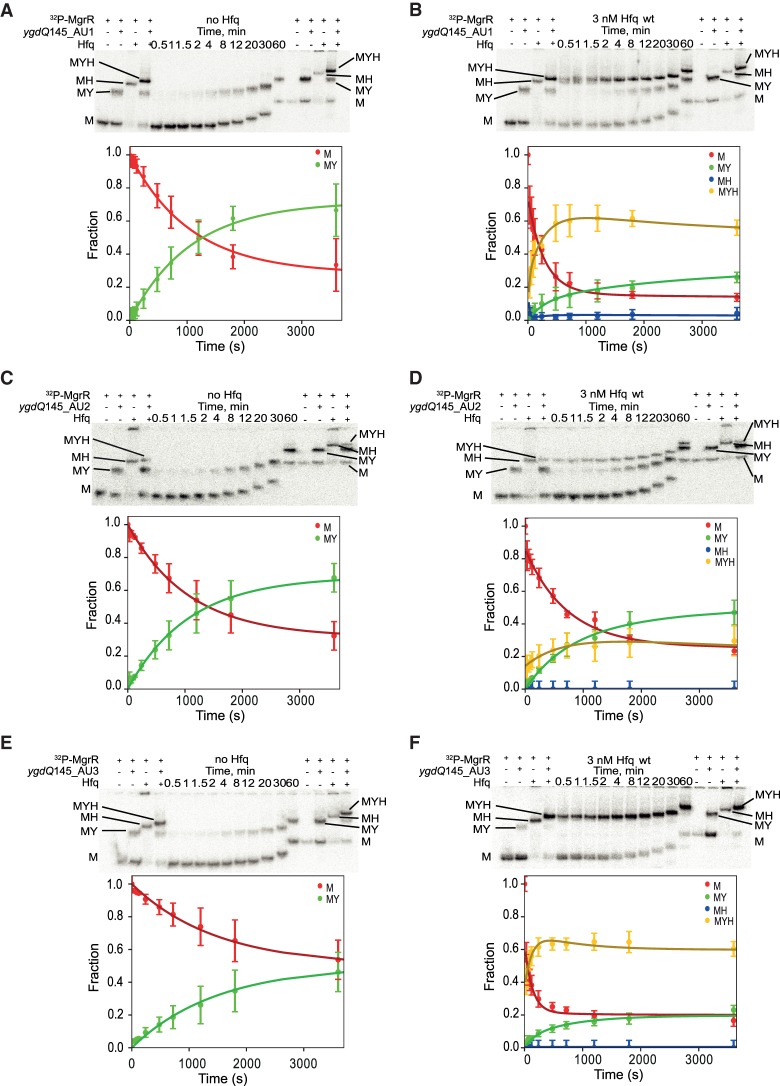
Global modeling of MgrR annealing to *ygdQ*145 with mutations in uridine-rich sequences. The native gel analysis of the kinetics of ^32^P-labeled MgrR annealing to (i) *ygdQ*145_AU1 in the absence of Hfq (*A*) or in the presence of Hfq (*B*), (ii) *ygdQ*145_AU2 in the absence of Hfq (*C*) or in the presence of Hfq (*D*), and (iii) *ygdQ*145_AU3 in the absence of Hfq (*E*) or in the presence of Hfq (*F*). The data for the annealing of MgrR to each of the mRNA mutants in the absence and presence of wt Hfq were analyzed globally, and the lines in the plots are the results of global fitting. The RNAs and their complexes are marked in the same way as in Figure 6.

To better understand how the Hfq protein contributes to the pairing of MgrR sRNA with *eptB* and *ygdQ*, several mutations were introduced into uridine-rich sequences of these mRNAs to test their importance for MgrR annealing (Figs. 10–13). In these experiments, the annealing of ^32^P-labeled MgrR to mRNA mutants was compared in the presence and absence of Hfq, and the two data sets were fit globally (Tables 5, 6; Figs. 11–13). Although not all rate constants could be uniquely derived, the fitting of the data to the model (Fig. 4) defined the essential changes induced by the mutations. Previous studies showed that when mutations were introduced at both the AU1 and AU2 sequence of *eptB*133, they detrimentally affected the regulation by MgrR in vivo (Schu et al. 2015). To test which of these uridine-rich sequences is more important for the Hfq-dependent annealing the same mutations were introduced into two mRNA mutants named *eptB*133_AU1_v1 and *eptB*133_AU2_v1 (Fig. 10A). The data showed that the mutations in the AU1 sequence motif did not markedly affect the annealing of MgrR to the *eptB*133_AU1_v1 mRNA either in the presence or absence of wt Hfq (Table 5; Fig. 11A,B). On the other hand, the mutations in the AU2 sequence motif resulted in lower accumulation of the ternary complex of ^32^P-MgrR with the *eptB*133_AU2_v1 mRNA and Hfq, and higher accumulation of the binary complex of MgrR with this mRNA mutant (Table 5; Fig. 11C,D). Additionally, these mutations induced about 10% accumulation of the binary MgrR-Hfq complex, which suggests that the mutations in the AU2 motif of *eptB*133 interfered in the formation of the ternary complex from the binary MgrR-Hfq complex and *eptB*133_AU2_v1 mRNA.

**TABLE 5.**
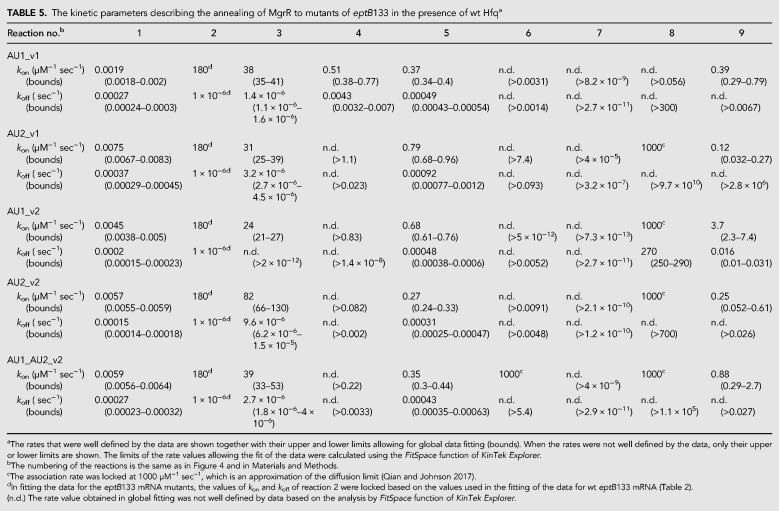
The kinetic parameters describing the annealing of MgrR to mutants of *eptB*133 in the presence of wt Hfq^a^

**TABLE 6.**
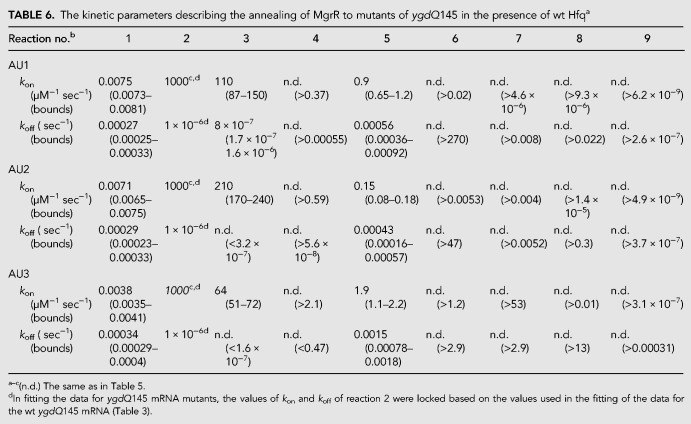
The kinetic parameters describing the annealing of MgrR to mutants of *ygdQ*145 in the presence of wt Hfq^a^

To test if a different set of mutations in the AU1 and AU2 sequence motifs would result in a similar effect, three additional *eptB*133 mRNA mutants were designed, which had mutations in the AU1 motif (*eptB*133_AU1_v2), the AU2 motif (*eptB*133_AU2_v2), or both (*eptB*133_AU1_AU2_v2) (Fig. 10A). The mutants were designed using *RNAstructure* program so as to minimize the interference into the secondary RNA structure (Reuter and Mathews 2010). As expected, the alternative mutations introduced into the AU1 sequence motif did not markedly affect the annealing of MgrR to the *eptB*133_AU1_v2 mRNA mutant (Table 5; Fig. 12A,B). On the other hand, the mutations in the AU2 sequence motif resulted in less efficient formation of the ternary complex, and higher fractions of binary complexes of MgrR with *eptB*133_AU2_v2, and binary complexes of MgrR with Hfq, in comparison to the AU1 mutant (Table 5; Fig. 12C,D). Finally, when mutations were introduced into both AU1 and AU2 sequence motifs their effect on the ternary complex formation was similar to when only the AU2 sequence was mutated (Table 5; Fig. 12E,F). Hence, these data support the importance of the uridine-rich sequence motif AU2 for the Hfq contribution to MgrR annealing to *eptB* mRNA, which explains the detrimental effect of mutations in this site for the regulation of *eptB* translation by MgrR in vivo (Schu et al. 2015).

To test the importance of uridine-rich sequences in the 5′-terminal region of *ygdQ* mRNA for MgrR annealing, mutations were introduced into the sequences AU1, AU2, and AU3 of *ygdQ*145 (Table 6; Figs. 10B, 13). The deletion of the 5′-terminal AU1 motif affected neither the direct association of MgrR to this mutant nor the ternary complex formation in the presence of Hfq, as compared to the reaction with wt *ygdQ*145 (Tables 3, 6; Figs. 9, 13A,B). On the other hand, the mutations introduced into the AU2 motif markedly decreased the formation of the ternary complex, leading to similar levels of accumulation of the ternary complex and the binary MgrR-*ygdQ*145_AU2 complex (Table 6; Fig. 13C,D). Finally, the mutation in the AU3 sequence did not interfere in the direct MgrR annealing to the *ygdQ*145_AU3 mutant nor in the formation of the ternary complex (Table 6; Fig. 13E,F). These data suggest that the uridine-rich AU2 sequence motif located 5′ to the MgrR binding site in *ygdQ* mRNA is involved in mRNA binding to Hfq, while the uridine-rich sequences either far upstream (AU1) or far downstream (AU3) of the MgrR binding site do not contribute to the MgrR pairing to *ygdQ* mRNA.

## DISCUSSION

### Non-canonical way of MgrR binding to Hfq

The results of competition studies showed that MgrR has strong affinities to both the proximal and the distal faces of Hfq enabling it to displace oligouridylates and oligoadenylates, which bind specifically to each site (Fig. 2). This result is consistent with previous observations that mutations in proximal and distal faces of Hfq decreased the stability of MgrR in turnover experiments in vivo (Schu et al. 2015). It is also consistent with the detrimental effects of K56A and Y25D Hfq mutations on the MgrR annealing to *eptB* and *ygdQ* mRNAs in vitro (Figs. 5, 6). On the other hand, when the equilibrium binding of MgrR to the Hfq mutants was studied in vitro, only the mutation in the proximal face showed a detrimental effect on binding (Supplemental Fig. S2). Indeed, although the Y25D mutation in the distal face of Hfq resulted in reduced accumulation of MgrR in vivo, and destabilized MgrR in rifampicin and inducer wash-out experiments, the removal of the A-rich sequence from MgrR did not affect the stability of MgrR in wash-out experiments with wt Hfq or Y25D Hfq mutants suggesting that the distal face interactions have lower contribution to the MgrR binding to Hfq in vivo than the proximal face interactions (Schu et al. 2015).

### The efficiency of MgrR in competition for Hfq

The data presented here show that MgrR sRNA was less efficient in competition for binding to Hfq than ChiX sRNA but more efficient than several other sRNAs (Table 1; Fig. 3). In agreement, it was previously reported that MgrR was among the most efficient sRNAs in competition against translation regulation of *rpoS* mRNA in bacterial cells (Moon and Gottesman 2011). It was also observed that although MgrR has a shorter lifetime than ChiX in in vivo turnover experiments with inducer washout, both are more stable than canonical sRNAs (Schu et al. 2015; Santiago-Frangos et al. 2016). Additionally, ChiX and MgrR more efficiently than other sRNAs induced β-galactosidase expression in bacterial three-hybrid assays designed to detect binding to Hfq (Berry and Hochschild 2018). Overall, these data suggest that MgrR and ChiX are more efficient in competition for Hfq than other sRNAs, in agreement with the data presented here (Table 1; Fig. 3). Interestingly, it was also observed that the competitive advantage of MgrR and ChiX over canonical sRNAs decreases when the Hfq C-terminal domain is removed, suggesting that additional contacts of these sRNAs with the distal face of Hfq are important to prevent dissociation induced by the proposed sweeping motion of the acidic C-terminal domain (Santiago-Frangos et al. 2016, 2017). Overall, these data suggest that the contribution of the distal face interactions to MgrR binding to Hfq improves its efficiency in competition for access to Hfq.

### The important role of the Hfq rim in MgrR annealing to its targets

Non-canonical binding of MgrR to Hfq (Fig. 2) explains the effects of mutations in RNA binding sites of Hfq on MgrR annealing to its targets (Figs. 8, 9) and on the MgrR stability in cells with the Hfq mutants (Schu et al. 2015). The mutations in the distal and proximal faces of Hfq resulted in lower accumulation of the ternary complexes of MgrR with each mRNA and Hfq, while the binary complexes of MgrR with the Hfq mutants were not detected (Figs. 8, 9). The effect of the distal face mutation was consistent with a slower formation of the ternary complex, and less efficient release of the binary MgrR-mRNA complex from Hfq, while the effect of the proximal face mutation was consistent with weaker binding of MgrR to the Hfq mutant (Figs. 8, 9, Tables 2, 3). These results support the explanation that the lower stability of MgrR in bacterial cells with these Hfq mutants was caused by the weaker protection of MgrR by Hfq from cellular RNases, because of less efficient formation of the complexes with Hfq (Schu et al. 2015). On the contrary, the mutation in the rim of Hfq trapped MgrR on Hfq and prevented its annealing to mRNA (Figs. 8, 9). This suggests that the binding of MgrR to both faces of the R16A Hfq mutant was not disturbed, while the mutation prevented mRNA binding to the Hfq rim, which prohibited the pairing. These results support the hypothesis that the increase in MgrR stability observed in cells with R16A Hfq mutants can be attributed to longer retention of MgrR on Hfq resulting from the decrease in its annealing to mRNA, and causing extended protection of MgrR from cellular RNases (Schu et al. 2015).

### Single-stranded, uridine-rich sequences in *eptB* and *ygdQ* mRNAs are used by Hfq to promote MgrR annealing

The RNA structure probing data showed that the A-rich sequence in MgrR and the U-rich sequences in *eptB* and *ygdQ* were located in single-stranded regions of the respective molecules (Fig. 1). These observations support the conclusion that even though the role of Hfq is to locally rearrange RNA structures, to do so it needs at least partially unstable structure regions available for its binding (Wroblewska and Olejniczak 2016). The sequence motifs involved in Hfq-dependent annealing of MgrR to *eptB* and *ygdQ* mRNAs are located upstream of the MgrR binding site in both mRNAs (Figs. 10–13), similarly as it was previously observed for canonical sRNAs (Panja and Woodson 2012). Interestingly, both in *eptB* and *ygdQ*, the sequences required for the role of Hfq in the annealing are mainly composed of uridine residues (Fig. 10). However, because the Hfq binding to these mRNAs is dependent on the interactions with the rim, which contains conserved arginine residues, it is likely that these interactions are mainly electrostatic. This would imply that their single-stranded character rather than a specific sequence is necessary for their efficient binding to the Hfq rim. Possibly, the use of uridine-rich sequences instead of adenosine-rich ones in mRNAs regulated by MgrR, could have evolved not because of the better binding of the uridine tracts to the rim of Hfq, but rather to prevent the binding of adenosine tracts to the distal site of Hfq, which would hinder the Hfq-dependent acceleration of Class II sRNA annealing.

## MATERIALS AND METHODS

### Preparation of RNA

sRNAs and fragments of *eptB* and *ygdQ* mRNAs were obtained using in vitro transcription with T7 RNA polymerase (Milligan et al. 1987). The templates for transcription were obtained by Taq polymerase extension of chemically synthesized overlapping oligodeoxyribonucleotides (Sigma-Aldrich) (Supplemental Table S1). After transcription, RNA molecules were purified using 8 M urea polyacrylamide gel electrophoresis as previously described (Olejniczak 2011). RNAs were 5′-^32^P-labeled using T4 polynucleotide kinase, which was followed by phenol–chloroform extraction, denaturing gel electrophoresis, and ethanol precipitation. 5′-^32^P-labeled RNAs were stored at −20°C as 200 nM solutions.

Chemically synthesized oligoribonucleotides U_18_ and A_27_ were kind gifts of Ryszard Kierzek (Institute of Bioorganic Chemistry, Polish Academy of Sciences).

### *E. coli* Hfq protein expression and purification

*E. coli* C-terminal His_6_-tagged Hfq protein was expressed from pET15b vector (Novagen) and purified as previously described (Małecka et al. 2015). Plasmids for expression of Hfq R16A, Hfq Y25D, and Hfq K56A mutants were generated from the pET15b-*hfq* vector using the QuikChange Site-Directed Mutagenesis Kit (Stratagene) with specific primers. After overexpression, cells were lysed by sonication. Then the lysate was applied to a HisTrap crude column (GE Healthcare) charged with NiSO_4_ and the sample was eluted with imidazole gradient. The preparation was treated with RNase A (30 μg/mL) and DNase I (5 U/mL) for 1 h at 37°C to remove contaminating nucleic acids. Then it was purified again on a Ni^2+^ affinity column. Eluted fractions were concentrated and loaded onto HiLoad 16/60 Superdex 200 size exclusion column (GE Healthcare). The sample was eluted in the storage buffer (50 mM HEPES pH 7.5, 250 mM NH_4_Cl, 1 mM EDTA, and 10% glycerol), and the Hfq concentration was determined from absorption at 280 nm as previously described (Olejniczak 2011).

### RNA structure probing

For RNA structure probing, RNAse T1 (Thermo Scientific), RNase T2 (Mo Bi Tec), and Nuclease S1 (Thermo Scientific) were used. Before the reactions with RNase T1 and RNase T2, 8 µL of 25 nM 5′-^32^P-labeled RNA was denatured by heating for 5 min at 65°C in a buffer composed of 12.5 mM Tris-HCl pH 7 and 125 mM KCl. Then, 1 μL of MgCl_2_ was added to a final concentration of 10 mM, followed by incubation for 5 min on ice. Before the reactions with Nuclease S1, 9 µL of 22 nM 5′-^32^P-labeled RNA was refolded in the same way as described above, in a buffer composed of 40 mM sodium acetate pH 4.5, 300 mM NaCl, and 2 mM ZnSO_4_. Probing reactions with each enzyme were initiated by adding 1 µL of respective RNase at indicated concentration. The final concentration of ^32^P-labeled RNA in the probing reactions was 20 nM and the total volume was 10 µL. Probing reactions with RNase T1 and Nuclease S1 were carried out for 10 min, and with RNase T2 for 15 min, at room temperature (RT). Blank samples were prepared in the same way, except that instead of enzyme 1 μL of water was added to the reaction.

To obtain an RNase T1 ladder, 20 nM 5′-^32^P-labeled RNA was incubated with 0.5 or 1 unit of RNase T1 in 7 M urea and 50 mM sodium citrate pH 4.3 for 10 min at 55°C, in a total volume of 10 µL. To obtain an alkaline hydrolysis ladder, a 33 nM final concentration of 5′-^32^P-labeled RNA was incubated in 83% formamide for 60 min at 100°C.

All reactions were stopped by adding 10 µL of STOP buffer (8 M urea, 20 mM EDTA). Samples were separated on 10% polyacrylamide gels (19:1 acrylamide/bis-acrylamide ratio) with 8 M urea in 1× TBE buffer. The gels were frozen and exposed to phosphor screens, then analyzed using a phosphorimager (Fujifilm FLA 500) with *ImageQuant* software.

### Competition assay

Competition assays were performed essentially as previously described (Małecka et al. 2015). Prior to use, RNAs were denatured in HBB buffer for 1 min at 90°C followed by incubation for 5 min on ice. In the reactions, 10 μL of 15 nM Hfq was mixed with 10 μL of 120 pM ^32^P-labeled A_27_ or ^32^P-labeled U_18_ and 10 μL of unlabeled RNA competitor at the required concentration range. Final concentrations were 5 nM Hfq and 40 pM ^32^P-A_27_ or ^32^P-U_18_. The reactions were incubated for 35 min at RT. Afterward, 25 μL aliquots were filtered and washed with 100 μL of 1× HBB. Membranes were dried and analyzed by phosphorimager after overnight exposure to phosphor screens. The data from competition experiments with unlabeled A_27_ and U_18_ were fit to the three-parameter equation F_B_ = B +(A − B)/(1 + 10^X−logIC50^), in which F_B_ is fraction bound, while A and B are the maximum and minimum fractions bound, respectively. The data from experiments with unlabeled MgrR were fit using the four parameter equation F_B_ = B +(A − B)/(1 + 10^(logIC50−X)×n^), where *n* is a Hill coefficient.

### Dissociation assay

The kinetics of sRNA displacement from Hfq was measured as previously described (Małecka et al. 2015). Prior to use, ^32^P-MgrR and unlabeled sRNAs were denatured separately in HBB for 1 min at 90°C, followed by 5 min incubation on ice. Then, 800 μL of 0.2 nM ^32^P-MgrR was mixed with 800 μL of 12 nM Hfq protein in HBB buffer and incubated for 20 min at RT. The dissociation reactions were initiated by mixing 125 μL of the binding reaction with 125 μL of 10 nM unlabeled sRNA. Final concentrations in the dissociation reactions were 0.05 nM ^32^P-MgrR, 3 nM Hfq, and 5 nM competitor sRNA. 17 μL reaction aliquots were filtered at indicated time points and washed with 100 μL of HBB. Membranes were dried, exposed to a phoshor screen, and analyzed by phosphorimager. The values of dissociation rates $k_{\mathrm{off}}^{1}$ and $k_{\mathrm{off}}^{2}$ were calculated from the data using segmental linear fitting with *GraphPad Prism* software as previously described (Olejniczak 2011; Małecka et al. 2015).

### Kinetics of sRNA–mRNA annealing

The kinetics of MgrR annealing to a 133-nt-long fragment of *eptB* mRNA, a 145-nt-long fragment of *ygdQ* mRNA, and their mutants, were measured using native electrophoretic mobility shift assay essentially as described before (Wroblewska and Olejniczak 2016). Prior to use, RNAs were refolded at 90°C for 1 min followed by incubation on ice for 5 min. For the reactions with ^32^P-labeled MgrR, the annealing reactions were initiated by mixing 4 nM ^32^P-labeled MgrR and 400 nM mRNA in the presence or absence of 12 nM Hfq hexamer in 1× HBB buffer. The final concentrations in the annealing reaction were 1 nM ^32^P-labeled MgrR, 100 nM mRNA, and 3 nM Hfq in a total volume of 64 µL. For the reactions with ^32^P-labeled *eptB*133 or ^32^P-labeled *ygdQ*145 mRNA fragments, the annealing reactions were initiated by mixing 4 nM ^32^P-labeled *eptB*133 or *ygdQ*145 with 400 nM MgrR in the presence or absence of 12 nM Hfq hexamer in 1× HBB buffer. The final concentrations in the annealing reactions were 1 nM ^32^P-labeled *eptB*133 or ^32^P-labeled *ygdQ*145, 100 nM unlabeled MgrR and 3 nM Hfq. During the annealing reaction, 5 μL aliquots were withdrawn at indicated times and loaded onto a running 6.5% polyacrylamide gel with TBM running buffer (89 mM Tris-HCl, 89 mM H_3_BO_3_, 2 mM MgCl_2_). The aliquots of control reactions were loaded immediately before the start of the annealing assay (controls incubated for 20 min), and immediately after the completion of the reaction (controls incubated for 80 min). The gels were dried (Bio-Rad Gel Dryer) and exposed to phosphor screens, followed by data quantification using a phosphorimager (Fujifilm FLA 5000) with *ImageQuant* software.

### Global data fitting of the kinetics of annealing

The annealing data were fit to a model shown in Figure 4, and described below, using *KinTek Explorer* software (Johnson et al. 2009b). The kinetic model was composed of the following reactions: (1) M + E = ME; (2) M + H = MH; (3) H + E = HE; (4) MH + E = MEH; (5) HE + M = MEH; (6) MEH + M = MEHM; (7) ME + MH = MEHM; (8) MEH + E = MEHE; (9) ME + HE = MEHE, where H is Hfq, M is MgrR, E is mRNA (either *eptB*133 or *ygdQ*145 in respective reactions), ME is a binary complex of MgrR with respective mRNA, MH is a binary complex of Hfq with MgrR, HE is a binary complex of Hfq with respective mRNA, MEH is a ternary complex of Hfq with MgrR and respective mRNA, MEHE is a complex of Hfq with MgrR and two molecules of respective mRNA, and MEHM is a complex of Hfq with respective mRNA and two molecules of MgrR.

The raw data quantified by phoshorimager analysis were used to calculate the fraction of each species observed during the reaction progress. The average of the data from at least three experiments was used in the fitting. The global analysis of the data for the annealing of MgrR to *eptB*133 or *ygdQ*145 in the presence of wt Hfq or mutants included four data sets describing the kinetics of annealing without Hfq, and with Hfq, when either MgrR or the respective mRNA was labeled while the alternate RNA was in 100-fold excess (see figure legends). The global analysis of the data for the annealing of MgrR to mutants of *eptB*133 or *ygdQ*145 mRNA fragments in the presence of wt Hfq included two data sets describing the kinetics of the annealing of ^32^P-labeled MgrR to a respective mRNA either without Hfq or with wt Hfq. In the reactions where the MH complex was not formed, for the fitting its fraction was assigned the value of zero and it was assigned the value of the standard deviation corresponding to the average for the reaction. The fitting of the data was followed by the confidence contour analysis using *FitSpace* program within *KinTek Explorer* to obtain the upper and lower limits (bounds) of individual rate constants that allow good fit to the data (Johnson et al. 2009a).

The kinetically preferred pathways in the reactions were assessed using *KinTek Explorer* by integration of the partial differentials for each species. For competing pathways, we reported the fraction of the integrated flux proceeding for each pathway (Table 4).

The free energy profiles for the competing pathways were computed using simple transition state theory, stating that the rate of reaction is a function of the activation energy. The exponential term below gives the fraction of molecules that have sufficient energy to reach the transition state. The transition state falls apart at the vibration frequency, defined by *k*_*B*_T/h. The following equations and constants were used:

$$k_{i}=\frac{k_{B}T}{h}exp\left(-\frac{\Delta G_{i}^{†}}{RT}\right),$$

$$\Delta G_{i}^{†}=RT\ln\frac{k_{B}T/h}{k_{i}},$$

$$k_{B}=1.38e^{-23}J/K,\;h=6.62e^{-34}Js,\;\frac{k_{B}T}{h}=6.21e^{12}s^{-1}\;\mathrm{at}\;25{}^{\circ}C,\;cal=4.184J,$$

where *k*_*B*_ is the Boltzman constant, *h* is Planck's constant, *k*_*i*_ is the observed rate constant, *R* is the gas constant, and *T* is the absolute temperature. Second-order rate constants were converted to pseudo-first-order rate constants using a nominal concentration of 10 nM as the standard state.

## SUPPLEMENTAL MATERIAL

Supplemental material is available for this article.

## Supplementary Material

Supplemental Material
