# Supplementary material for: The binding of Class II sRNA MgrR to two different sites on matchmaker protein Hfq enables efficient competition for Hfq and annealing to regulated mRNAs
Source: RNA. 2018 Dec;24(12):1761–84. doi: 10.1261/rna.067777.118 (PMC6239178; doi:10.1261/rna.067777.118)
Supplement: Supplemental Material [file supp_24_12_1761__index.html]

The binding of Class II sRNA MgrR to two different sites on matchmaker protein Hfq enables efficient competition for Hfq and annealing to regulated mRNAs — Supplemental Material 

# The binding of Class II sRNA MgrR to two different sites on matchmaker protein Hfq enables efficient competition for Hfq and annealing to regulated mRNAs

## Supplemental Material

- Supplemental\_data.docx
