# Supplementary material for: The binding of Class II sRNA MgrR to two different sites on matchmaker protein Hfq enables efficient competition for Hfq and annealing to regulated mRNAs
Source: RNA. 2018 Dec;24(12):1761–84. doi: 10.1261/rna.067777.118 (PMC6239178; doi:10.1261/rna.067777.118)
Supplement: Supplemental Material [file supp_067777.118_Supplemental_data.docx]

**SUPPLEMENTAL DATA for**

Joanna Mazur, Zuzanna Wroblewska, Kenneth A. Johnson, and Mikolaj Olejniczak *“The binding of Class II sRNA MgrR to two different sites on matchmaker protein Hfq enables efficient competition for Hfq and annealing to regulated mRNAs”*

**SUPPLEMENTAL MATERIALS AND METHODS**

**RNA structure probing**

The structure probing experiments were performed as described in the Materials and Methods in the main text.

**Equilibrium gel mobility shift assays**

Binding reactions were prepared by mixing 20 μL of ^32^P-MgrR (100 pM final concentration) with 20 μL of the Hfq dilutions in Hfq Binding Buffer (HBB) consisting of 24 mM Tris-HCl pH 7.5, 50 mM NaCl, 50 mM NH_4_Cl, 50 mM KCl, 0.5 mM EDTA and 5% glycerol (Lease and Woodson 2004) and incubated for 2 h at RT. Prior to use ^32^P-MgrR was renatured by heating at 90°C for 1 min followed by 5 min incubation on ice. For all reactions, 5 μl aliquots were loaded on running 5% native polyacrylamide gels in TBM running buffer (89 mM Tris-HCl, 89 mM H_3_BO_3_, 2 mM MgCl_2_), with acrylamide/bis-acrylamide ratio of 29:1. Gels were dried, exposed to phosphor screens, and quantified using Fujifilm phosphorimager with *ImageQuant* software. Experiments were performed at least in triplicate. The fraction of each species was measured by dividing the counts in each band by total counts in each lane. Equilibrium dissociation constant (*K*_d_) values were determined by fitting data to the quadratic equation:

$Y=Y_{0}+\Delta Y\frac{{[E]}_{0}+{[S]}_{0}+K_{d}-\sqrt{\left( {[E]}_{0}+{[S]}_{0}+K_{d} \right)^{2}-4{[E]}_{0}{[S]}_{0}}}{2{[E]}_{0}}$,

where *Y* is a fraction bound*,* *Y_0_* is bottom plateau, $\Delta Y$ is a difference between top and bottom plateaus. ${[E]}_{0}$ is concentration of ^32^P-labeled MgrR, which was 0.1 nM, and ${[S]}_{0}$ is the concentration of Hfq.

***References***

Lease, R. A. and S. A. Woodson (2004). "Cycling of the Sm-like protein Hfq on the DsrA small regulatory RNA." J Mol Biol **344**(5): 1211-1223.

**SUPPLEMENTAL TABLES**

**Supplemental Table S1. DNA oligonucleotides used in this study.**

| **Name** | **Sequence (5’ → 3’)** |
| --- | --- |
| chix_F | TAATACGACTCACTATAGGACACCGTCGCTTAAAGTGACGGCATAATAATAAAAAAATG |
| chix_R | AAAAAAATGGCCAATATCGCTATTGGCCCGTCAAAGAGGAATTTCATTTTTTTATTATTATGC |
| cyar_F | TAATACGACTCACTATAGGCTGAAAAACATAACCCATAAAATGCTAGCTGTACCAGGAACCACC |
| cyar_R | AAAAAATAAGCCCGTGTAAGGGAGATTACACAGGCTAAGGAGGTGGTTCCTGGTACAGCTAGC |
| dsra_F | TAATACGACTCACTATAGGAACACATCAGATTTCCTGGTGTAACGAATTTTTTAAGTGCTTCTTGC |
| dsra_R | AAAAAAATCCCGACCCTGAGGGGGTCGGGATGAAACTTGCTTAAGCAAGAAGCACTTAAAAAATTCG |
| mcas_F | TAATACGACTCACTATAGGACCGGCGCAGAGGAGACAATGCCGGATTTAAGACGCGGATGCACTGC |
| mcas_R | ATAAAAAAATAGAGTCTGTCGACATCCGCCAGACTCTACAGTACACACAGCAGTGCATCCGCGTCTT |
| mgrr_F | TAATACGACTCACTATAGGATTCGTTATCAGTGCAGGAAAATGCCTGTTAGCGTAAAAGCAAAACAC |
| mgrr_R | AAAAAAAACCGCCAGTAAACCGGCGGTGAATGCTTGCATGGATAGATTTGTGTTTTGCTTTTACGC |
| ryhb_F | TAATACGACTCACTATAGGCGATCAGGAAGACCCTCGCGGAGAACCTGAAAGCACGACATTGC |
| ryhb_R | AAAAAAAAAGCCAGCACCCGGCTGGCTAAGTAATACTGGAAGCAATGTGAGCAATGTCGTGCTTTCAGG |
| eptb133_F | TAATACGACTCACTATAGCGCGTGTAGATTTTACTTATCTGACTACCTCCGCACTTTTTCCCTGCCGGGCCTGAAAAGCCACTAAGCAGGG |
| eptb133_R | CTGTGTAATCGATTTGATGTATCTCATGCAAACAAACCCTGGACAAACAGGTGATAACACCCTGCTTAGTGGCTTTTC |
| eptb133_AU1_v1_F | TAATACGACTCACTATAGCGCGTGTAGACCGCACTTATCTGACTACCTCCGCACTTTTTCCCTGCCGGGCCTGAAAAGCCACTAAGC |
| eptb133_AU2_v1_F | TAATACGACTCACTATAGCGCGTGTAGATTTTACTTATCTGACTACCTCCGCACCCGCTCCCTGCCGGGCCTGAAAAGCCACTAAGC |
| eptb133_AU1_v1/AU2_v1_R | CTGTGTAATCGATTTGATGTATCTCATGCAAACAAACCCTGGACAAACAGGTGATAACACCCTGCTTAGTGGCTTTTCAGGCC |
| eptb133_AU1_v2_F | TAATACGACTCACTATAGCGCGTGTAGTTTTTTCTTTTCTGACTACCTCCGCACTTTTTCCCTGCCGGGCCTGAAAAGCCACTAAGCAGGG |
| eptb133 _AU2_v2_F | TAATACGACTCACTATAGCGCGTGTAGATTTTACTTATCTGACTACCTCCGCACCTCCTCCCTGCCGGGCCTGAAAAGCCAC |
| eptb133_AU1_AU2_F | TAATACGACTCACTATAGCGCGTGTAGATTCCACTCATCTGACTACCTCCGCACCTCCTCCCTGCCGGGCCTGAAAAGCCAC |
| eptb133_AU1_v2/AU2_v2/AU1_AU2_R | CTGTGTAATCGATTTGATGTATCTCATGCAAACAAACCCTGGACAAACAGGTGATAACACCCTGCTTAGTGGCTTTTCAGGCCCGGC |
| ygdq145_F | TAATACGACTCACTATAGCTTCATTTTTTTGGCAGGGCTTTTTAGATGTTATTTGCATGGATAACCGATCCTAACGCCTGGCTTGCGCTCGGTACGCTG |
| ygdq145_R | CTTTGCCACCACCAGAGAAAGGAAAATAATATTGTCGATCCCAAGAACGATCTCCAGCAGCGTCAGCGTACCGAGCGCAAGCCAG |
| ygdq145_AU1_F | TAATACGACTCACTATAGGCAGGGCTTTTTAGATGTTATATTTGCATGGATAACCGATCCTAACGCCTGGCTTGCGCTCG |
| ygdq145_AU2_F | TAATACGACTCACTATAGCTTCATTTTTTTGGCAGGGCTTCACAGATGTTATTTGCATGGATAACCGATCCTAACGCCTGGCTTGCGCTCGGTACGCTG |
| ygdq145_AU1/AU2_R | CTTTGCCACCACCAGAGAAAGGAAAATAATATTGTCGATCCCAAGAACGATCTCCAGCAGCGTCAGCGTACCGAGCGCAAGCCAGGCGTTAG |
| ygdq145_AU3_F | TAATACGACTCACTATAGCTTCATTTTTTTGGCAGGGCTTTTTAGATGTTATTTGCATGGATAACCGATCCTAACGCCTGGCTTGC |
| ygdq145_AU3_R | CTTTGCCACCACCAGAGAGAGGACCAGCACATTGTCGATCCCAAGAACGATCTCCAGCAGCGTCAGCGTACCGAGCGCAAGCCAGGCGTTAGGATC |

**SUPPLEMENTAL FIGURES**

Suplemental Figure S1

**
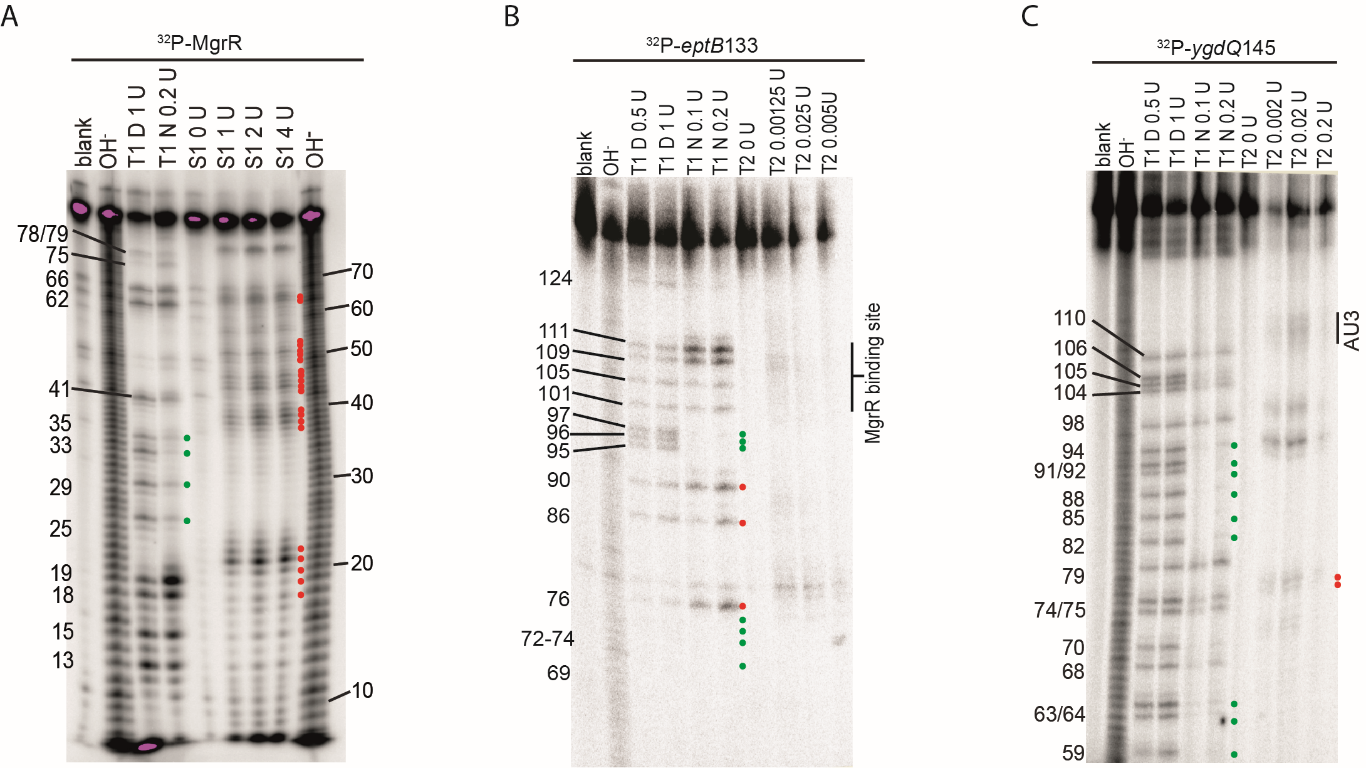
**

**Supplemental Figure S1. Probing of secondary structures of sRNA MgrR, and fragments of mRNAs *eptB*133 and *ygdQ*145.** (A) Probing of the structure of ^32^P-MgrR with RNase T1 and Nuclease S1. (B) Probing of the structure of ^32^P*-eptB*133 mRNA with RNases T1 and T2 using longer electrophoretic run for better separation of longer RNA fragments. (C) Probing of the structure of ^32^P*-ygdQ*145 mRNA with RNases T1 and T2 using longer electrophoretic run for better separation of longer RNA fragments. Symbols T1 D and T1 N denote probing with RNase T1 in denaturing or native conditions, respectively. The numbers to the left indicate positions of guanosine residues. Blank denotes untreated control and OH^-^ denotes formamide ladder. Positions of the residues constrained as single-stranded in structure prediction using *RNAStructure* software (Figure 1 in main text) are marked with red dots, and those constrained as double-stranded with green dots.

Supplemental Figure S2


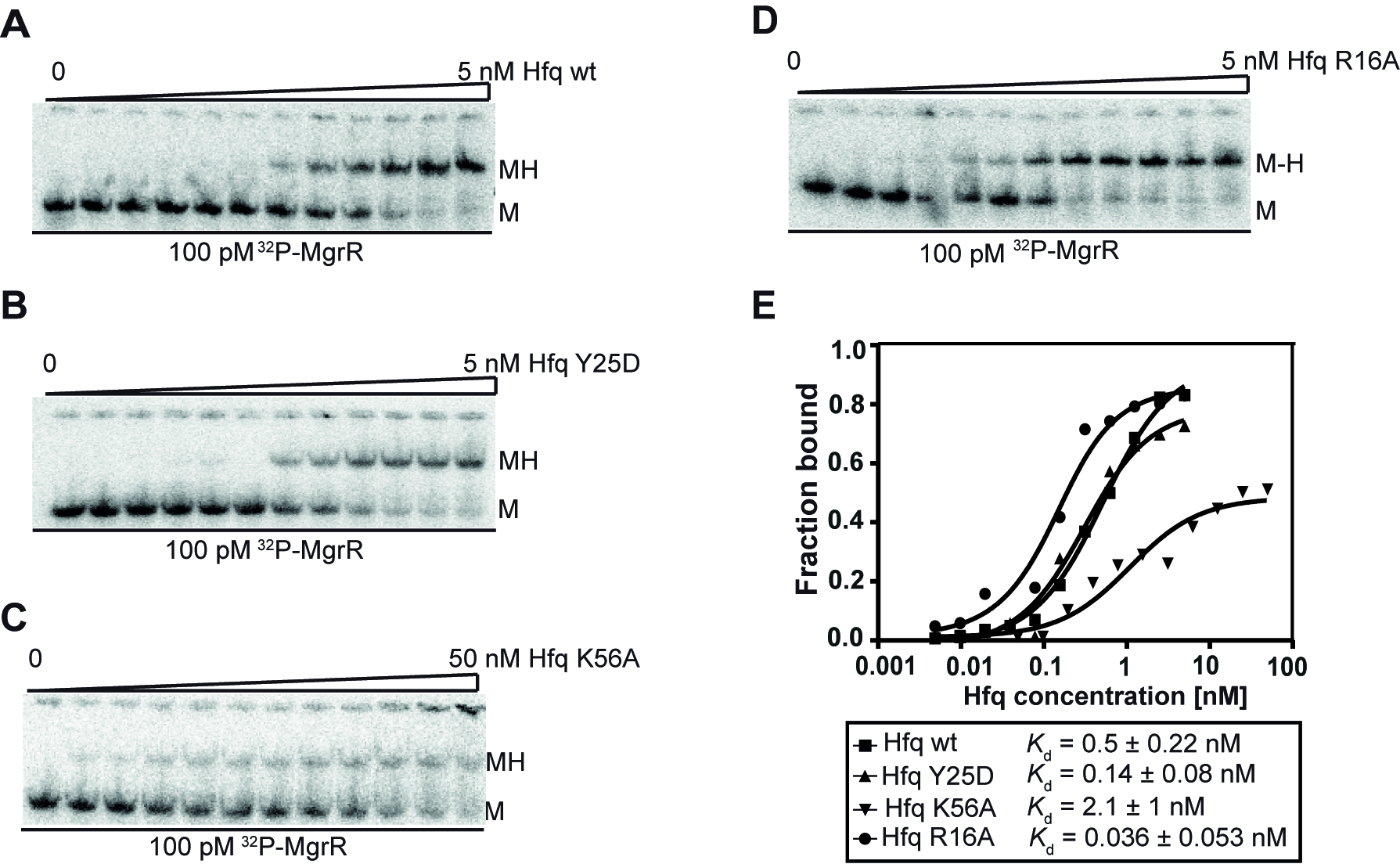


**Supplemental Figure S2. Equilibrium binding of MgrR sRNA to the Hfq protein and its mutants**. (A-D) The binding of ^32^P-MgrR to Hfq and its mutants monitored using gelshift assay. Concentrations of the Hfq protein are indicated above the gels. Symbols M and M-H refer to free MgrR or MgrR in complex with Hfq, respectively. (E) The fitting of data from A-D using the quadratic equation provided *K*_d_ values of 0.48 nM for Hfq wt, 0.26 nM for Hfq Y25D, 1 nM for Hfq K56A and 0.09 nM for Hfq R16A. Average equilibrium dissociation constant (*K*_d_) values for MgrR binding to Hfq and its mutants are shown in the legend. The *K*_d_ values are averages from experiments done at least in triplicate.
